# Understanding signatures of positive natural selection in human zinc transporter genes

**DOI:** 10.1038/s41598-022-08439-y

**Published:** 2022-03-12

**Authors:** Ana Roca-Umbert, Rocio Caro-Consuegra, Diego Londono-Correa, Gabriel Felipe Rodriguez-Lozano, Ruben Vicente, Elena Bosch

**Affiliations:** 1 Institut de Biologia Evolutiva (UPF-CSIC), Departament de Medicina i Ciències de la Vida, Universitat Pompeu Fabra, Parc de Recerca Biomèdica de Barcelona, 08003 Barcelona, Spain; 2 Laboratory of Molecular Physiology, Universitat Pompeu Fabra, Parc de Recerca Biomèdica de Barcelona, 08003 Barcelona, Spain; 3 Centro de Investigación Biomédica en Red de Salud Mental (CIBERSAM), 43206 Reus, Spain

**Keywords:** Evolution, Evolutionary genetics, Population genetics, Evolutionary biology, Genetic variation

## Abstract

Zinc is an essential micronutrient with a tightly regulated systemic and cellular homeostasis. In humans, some zinc transporter genes (ZTGs) have been previously reported as candidates for strong geographically restricted selective sweeps. However, since zinc homeostasis is maintained by the joint action of 24 ZTGs, other more subtle modes of selection could have also facilitated human adaptation to zinc availability. Here, we studied whether the complete set of ZTGs are enriched for signals of positive selection in worldwide populations and population groups from South Asia. ZTGs showed higher levels of genetic differentiation between African and non-African populations than would be randomly expected, as well as other signals of polygenic selection outside Africa. Moreover, in several South Asian population groups, ZTGs were significantly enriched for SNPs with unusually extended haplotypes and displayed SNP genotype-environmental correlations when considering zinc deficiency levels in soil in that geographical area. Our study replicated some well-characterized targets for positive selection in East Asia and sub-Saharan Africa, and proposes new candidates for follow-up in South Asia (*SLC39A5*) and Africa (*SLC39A7*). Finally, we identified candidate variants for adaptation in ZTGs that could contribute to different disease susceptibilities and zinc-related human health traits.

## Introduction

After the Out of Africa event around 60–70 kya, modern humans spread across the world, encountering multiple new environments and adopting different lifestyles^1^. Human populations arriving in newly colonized regions had to change their diets and adapt to the locally available food resources and nutrients. Thus, the environmental diversity encountered during the human expansions from Africa, together with the lifestyle changes experienced in recent millennia, forced modern human populations to cope with significant dietary modifications in both time and space. As a result of such new selective pressures, local genetic adaptations to nutrient availability and food resources likely arose in different human groups, leaving molecular signatures in the genomes of present-day populations^2,3^.

Many studies sought to identify and understand the genetics behind our adaptation to local selective pressures related to diet. Some of the most well-known cases are associated with the metabolism of macronutrients in the human body and include genetic adaptations to dairy consumption, high-fat diets, and starchy food, among others^4–6^. However, other research indicates that alterations in our dietary intake of micronutrients, such as selenium and zinc, may have also exerted strong selective pressures on humans, which could explain the high levels of population differentiation and signals of positive selection detected in genes related to the transport and metabolism of micronutrients^7,8^. For instance, the C282Y allele at the *HFE* gene, which contributes to hemochromatosis, was suggested to have been selectively advantageous during the Neolithic in Europe, as it favored iron absorption in an environment deficient in this micronutrient^9^. Nevertheless, genetic surfing has also been suggested as an equally reasonable explanation for the C282Y allele frequency gradient observed across Europe^10^. Similarly, genetic variation in the *AS3MT* gene has been reported to protect native populations from South America from the toxic arsenic-rich water in their habitat^11^.

Micronutrients are vitamins and minerals that in very low amounts are fundamental for maintaining health: most cannot be produced by the organism and must be obtained through diet^12,13^. Notably, zinc is one of the most ubiquitous trace elements in humans, where it is required for the correct function of numerous enzymes, transcription factors, and signaling molecules. Moreover, zinc is involved in several biological mechanisms with different structural, catalytic, and regulatory roles^14–16^. Several studies have shown that zinc deficiency in humans affects multiple physiological and metabolic processes, leading to growth retardation, a dysfunctional immune response, and even cognitive impairment^14,17,18^. In turn, the worldwide distribution of zinc in soils is quite diverse^19,20^, a low content easily resulting in zinc deficiency in living organisms^16^. Thus, in some regions of the world human zinc deficiency has been recognized as a nutritional problem with life-threatening consequences; South Asia, and India in particular, are among the most severely affected^16,19,21,22^.

As a healthy organism depends on the maintenance of correct zinc levels, the systemic and cellular homeostasis of this micronutrient needs to be closely regulated. In humans, the main proteins responsible for this homeostasis are 24 zinc transporters, which are located at specific tissues and organelles^14,23^. They are divided into two groups: the ZnT family, which imports zinc into the cytosol, and the ZIP family, which exports zinc out of the cell or into the cell organelles. The ZnT family is encoded by 10 genes named *SLC30A1*-*10*, and the ZIP family by 14 genes named *SLC39A1-14*, collectively referred to as Zinc Transporter Genes (ZTGs)^14,24–26^. Zinc transporters are known to play a role in several human diseases, such as cancer, immunodeficiency, diabetes, and neurodegenerative diseases, as reviewed by various authors^23,27,28^.

Previous scans of positive selection have identified some individual ZTGs as strong candidates for adaptation in different reference populations from worldwide datasets. For instance, the *SLC30A9* gene shows signatures of selection in Asian populations^29–32^, *SLC39A8* in the East Asian Han Chinese from Beijing^33^, and *SLC39A4* in sub-Saharan Africa^34–36^. As expected under a hard selective sweep model, these candidates were found among the top genome-wide departures from neutral deviations when exploring the site frequency spectrum, unusually extended haplotypes, and/or extreme patterns of population differentiation. In turn, it has been suggested that differences in zinc homeostasis among populations might be adaptive to local dietary and environmental conditions. In accordance with this, extreme patterns of population differentiation have also been found for different functional variants in ZTGs^8,33^. Moreover, a strong correlation has been described between the zinc content in cropland and the frequency of a putatively selected haplotype at the *SLC30A9* gene^8^, whereas the extremely high frequency of the Val372 isoform encoded by *SLC39A4* in sub-Saharan Africa was suggested to provide resistance to certain pathogens by starving them of zinc^36^. However, since human zinc homeostasis arises from the complementary action of 24 zinc transporters, other more subtle forms of selection could also have facilitated human adaptation to zinc availability through concerted allele frequency shifts across the complete set of ZTGs. Indeed, it has been recognized that although the so-called classic selective sweeps might be generally rare in recent human history^37^, polygenic adaptation might have had a more important role in facilitating rapid adaptation to new environmental selective pressures^38,39^. However, to capture the genomic signatures of polygenic selection, other statistical strategies might be required: for example, gene set enrichment approaches that test whether a particular set of loci are enriched among the top selection scores, or alternative methods that aggregate the putative signals of positive selection across the multiple loci contributing to the targeted adaptive phenotype^40^.

Here, we study the potential impact of zinc availability on human adaptation by exploring the molecular signatures left by natural selection on the complete set of ZTGs. To do so, we not only compiled evidence for the classic hard selective sweep model but also focused on testing whether the complete set of ZTGs are enriched for other more subtle signals, as expected under a polygenic scenario (that is, when selection may have acted simultaneously on more than one gene). We therefore first analyzed global patterns of ZTG variation using whole-genome data from Phase 3 of the 1000 Genomes Project^41^, which comprises human populations from the five main continental regions. We then extended this analysis to different population groups from South Asia using whole-genome data from the Pilot phase of the GenomeAsia 100 K Project^42^ to investigate a specific geographical region with well-recognized zinc deficiency^22^. As an additional signal of selection in this area, we also explored the environmental correlation between SNP genotypes in ZTGs and levels of zinc deficiency in Indian soils. Our results show that the complete set of ZTGs presents extreme levels of genetic differentiation between African and non-African populations, as well as unusual patterns of genetic variation arising from different strong geographically restricted selective sweeps across a few ZTGs, and other more subtle forms of adaptation, especially in South Asia. Finally, we highlight several candidate variants for selection in the ZTGs, some of them correlating with zinc soil availability, which deserve further functional studies and experimental validation.

## Results

### Datasets and populations

**Figure 1:**
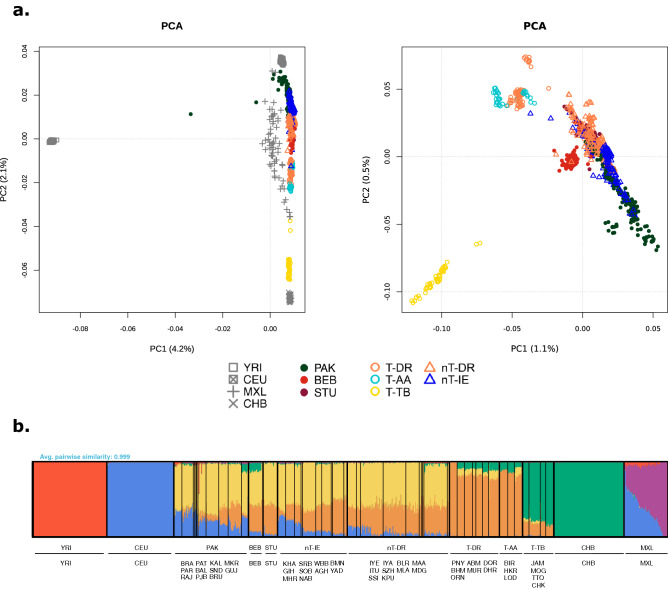
Population structure analysis of the South Asian dataset. (**a**) Principal Component Analysis (PCA) of the curated dataset with (left) and without (right) external reference populations. (**b**) ADMIXTURE analysis of the curated dataset, K = 6 (CV error = 0.4274). For populations from the 1000 GP belonging to the South Asian region, a subset of 15 samples is represented. Population group abbreviations: *T-AA* tribal populations speaking Austroasiatic languages, *T-DR* Dravidian-speaking tribal populations, *T-TB* Tibeto-Burman-speaking tribal populations, *nT-DR* non-tribal populations speaking Dravidian languages, *nT-IE* non-tribal populations speaking Indo-European languages, *PAK* Pakistan, *BEB* Bangladesh, *STU* Sri Lanka, *YRI* Yoruba in Ibadan, Nigeria, Africa, *CEU* Utah residents with Northern and Western European ancestry, *CHB* Han Chinese in Beijing, China, *MXL* individuals with Mexican ancestry from Los Angeles, California. The full names for all populations within groups are available in Supplementary Table S3.

We first compiled whole-genome sequencing data to analyze the patterns of variation in ZTGs on two geographical levels. Thus, we explored a worldwide dataset of 2,328 unrelated individuals representing 24 populations across Africa (AFR), Europe (EUR), East Asia (EAS), South Asia (SAS) and America (AMR), denoted as the 1000GP dataset (for details, see “Methods” section, Supplementary Table S1). We also gathered a South Asian dataset to further describe ZTG variation in a geographical region with well-recognized zinc deficiency in soil^19^ (for details, see Supplementary Table S2). After PCA and ADMIXTURE analyses, eight genetic homogenous population groups with sample sizes ≥ 30 from this latter dataset were selected to be analyzed for signatures of positive selection (Fig. 1, Supplementary Figs. S1, S2). Thus, the final curated South Asian dataset comprised a total of 1353 individuals (979 of them South Asian) and included four external reference populations (Yoruba in Ibadan, Nigeria (YRI); Utah residents with Northern and Western European ancestry (CEU); Han Chinese in Beijing, China (CHB); and individuals with Mexican Ancestry (MXL)), populations from Pakistan (PAK), Bangladesh (BEB) and Sri Lanka (STU), and five Indian groups: tribal populations speaking Austroasiatic languages (T-AA), Dravidian-speaking tribal populations (T-DR), Tibeto-Burman-speaking tribal populations (T-TB), non-tribal populations speaking Dravidian languages (nT-DR), and non-tribal populations speaking Indo-European languages (nT-IE) (Fig. 1, Supplementary Table S3).

### Population differentiation in ZTGs

**Figure 2:**
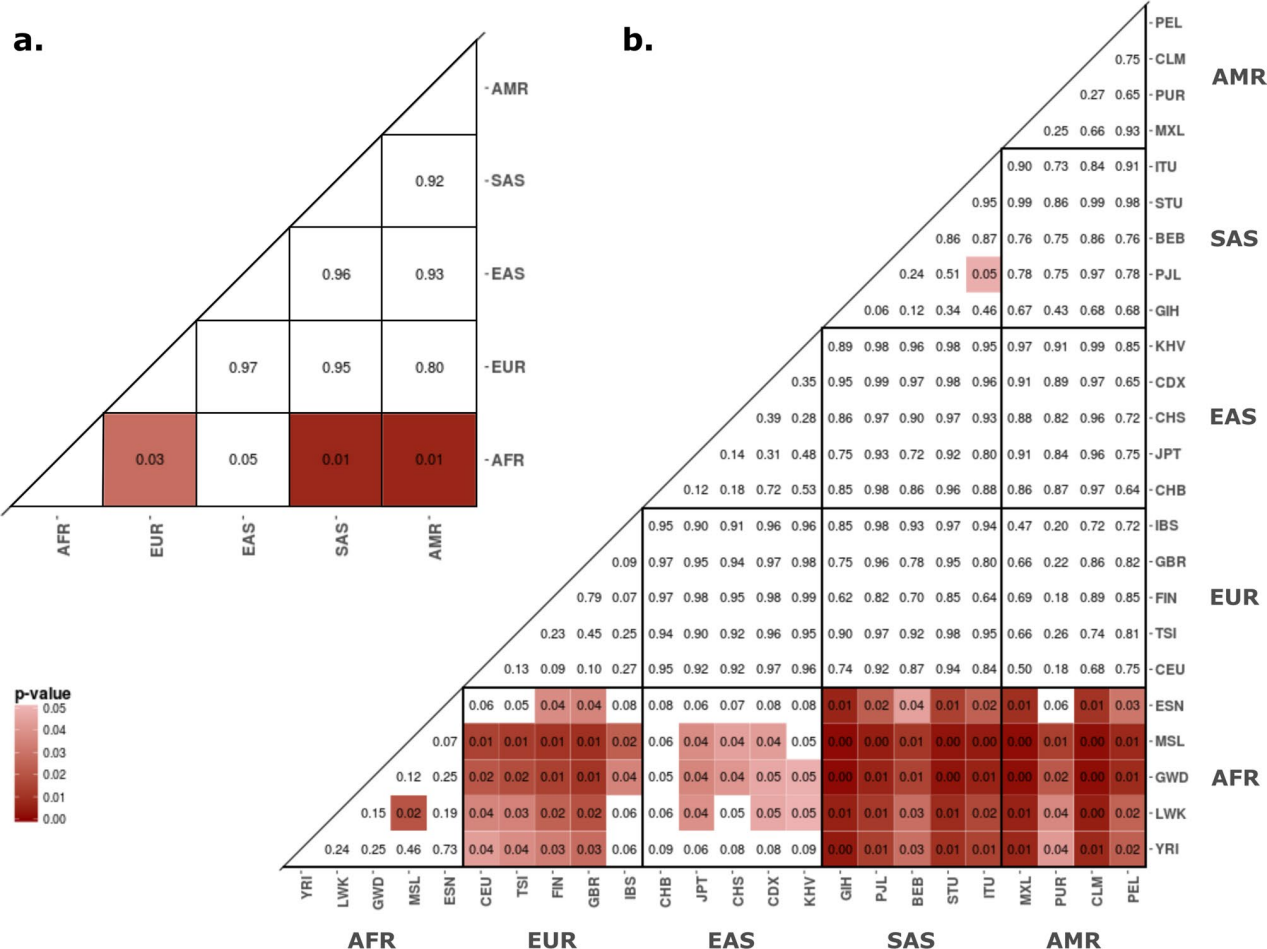
ZTGs present higher differentiation than randomly matched genes in African versus non-African comparisons. (**a**) For each F_ST_ comparison between geographical regions in the 1000GP dataset, p-values of the corresponding rank test when comparing the average WA F_ST_ value of the whole set of 24 ZTGs with that of 10,000 resamplings of 24 genome-wide matched genes. (**b**) For each F_ST_ population comparison in the 1000GP dataset, p-values of the corresponding rank test when comparing the average WA F_ST_ value of the whole set of 24 ZTGs with that of 10,000 resamplings of 24 genome-wide matched genes.

We investigated the patterns of genetic differentiation of the complete set of ZTGs, comparing each of the 24 worldwide populations and the five main geographical regions of the 1000GP dataset. For that, we computed SNP F_ST_ values for all pairs of populations and geographical regions in the 1000GP dataset and extracted both the highest F_ST_ value (Max F_ST_) and the weighted average F_ST_ value (WA F_ST_) per gene. Subsequently, a rank test was used to assess whether the mean WA F_ST_ (and mean Max F_ST_) of the complete set of ZTGs differed from genome-wide expectations using 10,000 resamplings of 24 randomly matched genes (for details, see “Methods” section, Supplementary Figs. S3, S4). Notably, the ZTGs showed a consistent pattern of higher WA F_ST_ (and higher Max F_ST_) than random genome-wide gene sets in all continental pairwise comparisons with Africa, except for East Asia (Fig. 2, Supplementary Fig. S5). Moreover, several individual populations within each geographical region clearly replicate the high genetic differentiation of ZTGs when compared with African populations (Fig. 2, Supplementary Fig. S5, Table S5). Similarly, a greater proportion of highly differentiated SNPs was found in ZTGs when compared to sets of randomly matched genes in several African versus non-African population comparisons. However, in the comparison of global geographical regions, a greater proportion of highly differentiated SNPs in ZTGs was only detected when comparing East Asia and Africa (Supplementary Fig. S6, Table S5). As for the South Asian dataset, ZTGs had a greater proportion of highly differentiated SNPs than random genes in two groups of Indian tribal populations (T-DR and T-TB) when compared with the YRI population. In contrast, analysis of Max F_ST_ and WA F_ST_ did not reveal any greater genetic differentiation in the complete set of ZTGs when the South Asian and YRI populations were compared (Supplementary Table S6).

The potential contribution of each individual ZTG to the genetic differentiation in the whole gene set was then examined by (i) comparing the F_ST_ of each ZTG with the corresponding genome-wide distribution of F_ST_ gene values obtained from 5146 reference genes (for details, see “Methods” section, Supplementary Fig. S4), and (ii) for the 1000GP dataset, testing the genetic differentiation of the complete set of ZTGs with the same procedures as described above but removing one of the 24 ZTGs each time. For most African versus non-African comparisons in the 1000GP dataset, *SLC30A9* and *SLC39A5* appeared as clear outliers in the WA F_ST_ analysis, whereas *SLC39A4*, *SLC39A11,* and again *SLC30A9* were the most consistent outliers when considering the Max F_ST_ per gene. *SLC39A11* also appeared as a Max F_ST_ outlier in several non-African pairwise comparisons as well as when comparing the geographical regions South Asia and Europe (Supplementary Table S5). The removal of any individual ZTG in the 1000GP dataset did not affect the general trend of higher differentiation in ZTGs when comparing African with non-African populations (results not shown) but confirmed that *SLC30A9* was clearly the gene contributing most to the observed pattern (for the effects of its removal, see Supplementary Fig. S7). In the South Asian dataset, *SLC30A9* and *SLC39A5* were replicated as the two main outliers in the WA F_ST_ analysis, while *SLC30A9* and *SLC39A11* were consistent outliers in several of the South Asian groups when considering the Max F_ST_ per gene (Supplementary Table S6). However, the Max F_ST_ signal for *SLC39A4* was not replicated because the SNP responsible was filtered out during the merging of the South Asian dataset.

### Other signals of positive selection in ZTGs

Additional signatures of positive selection were explored using the cross-population Extended Haplotype Homozygosity (XP-EHH) and the integrated Haplotype Score (iHS) statistics as well as the Tajima’s D test. As above, we first tested whether the distribution of the statistics computed across the whole set of 24 ZTGs differed from genome-wide randomly matched genes and then determined which individual ZTGs contributed most to the signals.

In the 1000GP dataset, we detected higher Max XP-EHH values in ZTGs than in randomly matched genes in three (CEU, GIH, and MXL) out of the four non-African populations analyzed when using YRI as the reference population. Moreover, the signature of higher Max XP-EHH values observed for the whole set of ZTGs in the GIH population was replicated when using CHB as the reference population. For CEU (and CHB) we also observed a higher proportion of SNPs with outlier XP-EHH values when using MXL as the reference (Supplementary Table S7). However, we did not detect higher Avg XP-EHH values in ZTGs in any of these 1000GP dataset populations. *SLC30A9*, *SLC30A10*, *SLC39A8,* and *SLC39A11* were the ZTGs that most contributed to the Max XP-EHH signal (Supplementary Table S7)*.* In agreement with the XP-EHH results in the GIH population, ZTGs had consistently higher proportions of SNPs with outlier XP-EHH values than randomly matched genes in four South Asian groups (i.e., BEB, nT-DR, nT-IE, and PAK) when using CHB as the reference. As previously observed in the GIH population, *SLC30A10* and *SLC39A11* were the ZTGs that most contributed to the outlier Max XP-EHH signals in these South Asian groups (Supplementary Table S8).

Interestingly, higher iHS values were obtained for ZTGs than for genome-wide randomly matched genes, but only in some East and South Asian populations of the 1000GP dataset (Supplementary Table S9). In particular, ZTGs had higher Avg iHS values in GIH and STU, as well as higher Max iHS in GIH, STU, PJL, and KHV. Notably, the mean Max iHS across the 24 ZTGs in these populations ranged from 2.12 to 2.22, |iHS|> 2 being the threshold usually considered as evidence of recent positive selection at a given locus^43^. When analyzing the genes contributing most to the iHS signatures, *SLC30A9* was a clear outlier for Avg iHS, whereas *SLC30A10* and particularly *SLC39A11* were the genes that most consistently contributed to the increased Max iHS values of ZTGs. In the GIH population, we also detected a higher proportion of top 1% iHS values for the complete set of ZTGs when compared to randomly matched genes and up to three further individual ZTGs contributing to the Max iHS signature of ZTGs (*SLC30A9, SLC39A10*, and *SLC39A12*; Supplementary Table S9). Accordingly, most population groups of the South Asian dataset showed consistently higher iHS values across the complete set of 24 ZTGs compared to randomly matched genes (Table 1). Notably, in the nT-IE population group and the PAK and STU populations, ZTGs displayed not only a higher proportion of SNPs with outlier iHS values but also higher Max iHS and Avg iHS values than randomly matched genes. As above, *SLC30A9* was the ZTG contributing most to the Avg iHS signal, while *SLC30A9*, *SLC30A10*, *SLC39A10,* and *SLC39A11* were found to consistently contribute to the Max iHS signature detected in the South Asian dataset. Moreover, the Max iHS value in these outlier genes was always ≥ 3.19 (Supplementary Table S10).

**Table 1:** ZTGs tend to have higher iHS values than randomly matched genes in several South Asian population groups.

	T-AA		T-DR		T-TB		BEB		nT-DR		nT-IE		PAK		STU	
	│iHS│	p-value	│iHS│	p-value	│iHS│	p-value	│iHS│	p-value	│iHS│	p-value	│iHS│	p-value	│iHS│	p-value	│iHS│	p-value
Prop SNP		0.407		0.014*		0.077		0.061		0.014*		0.002**		0.017*		0.042*
Max iHS	1.87	0.215	1.99	0.198	1.82	0.355	2.1	0.051	2.11	0.067	2.24	0.013*	2.2	0.012*	2.15	0.022*
Avg iHS	0.90	0.042*	0.89	0.137	0.77	0.457	0.96	0.014*	0.96	0.027*	1.01	0.005**	0.98	0.009**	0.99	0.005**

*Prop SNP* analysis for an unusual proportion of SNPs with top 1% |iHS| values, *Max iHS* analysis considering the mean maximum |iHS| value per gene across the whole set of 24 ZTGs, *Avg iHS* analysis considering the mean average |iHS| value per gene across the whole set of 24 ZTGs. *T-AA* tribal populations speaking Austroasiatic languages, *T-DR* Dravidian-speaking tribal populations, *T-TB* Tibeto-Burman-speaking tribal populations, *BEB* individuals from Bangladesh, *nT-DR* non-tribal populations speaking Dravidian languages, *nT-IE* non-tribal populations speaking Indo-European languages, *PAK* populations from Pakistan, *STU* individuals from Sri Lanka.

*p-value < 0.05; **p-value < 0.01. Note: the Max iHS signal was replicated with the SUMSTAT statistic (nT-DR, p-value = 0.047; nT-IE, p-value = 0.017; PAK, p-value = 0.003 and STU, p-value = 0.011) (for more details, see Supplementary Table S10).

When exploring the site frequency spectrum of the whole set of ZTGs across the 24 populations of the 1000GP dataset, we found no enrichment of ZTGs towards Tajima’s D negative values compared to genome-wide randomly matched genes (Supplementary Table S11). However, some of the ZTGs that contribute to the higher XP-EHH and iHS signals detected in ZTGs were also the top outlier genes in the genome-wide distribution of Tajima’s D values obtained with all 5146 reference genes. For instance, *SLC39A5* was detected as a clear Tajima’s D outlier in most non-African populations, especially in those of South Asia, whereas *SLC30A9* was only found as an outlier for negative values of Tajima’s D in some East Asian populations. Finally, *SLC39A7* was detected as a consistent Tajima’s D outlier for negative values in all African populations (Supplementary Table S11).

### Replicating evidence for polygenic adaptation in ZTGs

We also used the SUMSTAT test^44^ to assess whether the sum of the max gene scores of all 24 ZTGs for each statistic replicated their corresponding enrichments in signals of positive selection when compared to randomly matched genes, while also controlling for SNP density as in Daub et al.^40^. Although this approach was much more statistically stringent, the complete set of ZTGs retained a clear enrichment for higher population differentiation in several African versus non-African population comparisons (Europe, South Asia, and America; Supplementary Fig. S8), and for stronger Max iHS signals in the STU population of the 1000GP dataset (Supplementary Table S9) as well as the C-DR, C-IE, PAK and STU populations of the South Asian dataset (Table 1, Supplementary Table S10). On the contrary, although we detected higher average Max XP-EHH values across ZTGs compared to randomly matched genes in CHB, GIH and MXL, no enrichment for Max XP-EHH signals was replicated with the SUMSTAT test (Supplementary Tables S7, S8).

### Identification of candidate genes and variants for selection

We then looked for individual ZTGs that were recurrently identified as outliers across the F_ST_, XP-EHH, iHS, and Tajima’s D analyses in comparison with reference genes matched for gene length, recombination, and gene content (Supplementary Tables S5–S7). Out of the 24 human ZTGs, six displayed consistent patterns of variation indicative of strong positive selection across several populations in distinct geographical regions. As expected, these outlier ZTGs comprise previously identified targets for selection in East Asia (*SLC30A9*, *SLC39A8*)^8,33^, sub-Saharan Africa (*SLC39A4*)^36^, or found to be widespread across continents (*SLC39A11*)^8^. However, we also identified two additional ZTGs with distinctive levels of population differentiation, deviations in the site frequency spectrum, and unusually extended haplotypes, which are therefore proposed as new putative candidates for positive selection in Africa (*SLC39A7*) and South Asia (*SLC39A5*) (for details, see Supplementary Note 1).

Furthermore, we also explored the contribution of individual SNPs to the specific patterns of population differentiation and signals of positive selection detected in the ZTGs. For that, we examined the SNP values for each statistic and focused on those with a score above the 99th percentile in at least 95% of the 10,000 resampling sets of 24 randomly matched genes in each individual population or population comparison analyzed (Supplementary Fig. S4). After their corresponding annotation, we selected as candidate SNPs for positive selection those that presented at least one indicator of functionality and/or evolutionary conservation (complete lists for each dataset are provided in Supplementary Tables S12, S13).

Among the genetic variants contributing the most to the extreme African versus non-African differentiation in the *SLC30A9* gene, we found many linked eQTLs, one non-synonymous SNP (rs1047626), and five SNPs with CADD Phred Scores greater than 12 (rs2660319, rs15857, rs55835604, rs4861014, rs7660233). The greatest allele frequency differences for these candidate SNPs are found between East Asia and Africa, whereas the intermediate frequencies observed in the South Asian population groups probably explain and allow the capture of the same adaptive event with the iHS statistic. Although we detected no obvious candidate SNP for *SLC39A5* and *SLC39A7*, for *SLC39A11* we identified two intronic SNPs with CADD Phred Scores greater than 12 (rs6501559, rs8068946) and several eQTLs presenting extreme allele frequency differences between African and non-Africans. Similarly, most of the *SLC39A8* outlier XP-EHH signals identified in CHB (when using MXL as the reference) and several of the SNPs unusually differentiated between some East Asian and South Asian populations were also identified as eQTLs for the gene. At the *SLC30A10* region, we identified several eQTLs highly differentiated between GWD and CLM and contributing to the iHS signal detected in Europe and South Asia, but also variants at an intronic ncRNA producing the XP-EHH signatures detected in Europe and in several South Asian groups (when using CHB as the reference). Furthermore, several eQTLs for *SLC30A2*, *SLC30A8*, *SLC39A3*, *SLC39A6*, *SLC39A9*, *SLC39A10*, *SLC39A12,* and *SLC39A14* were identified as additional candidate SNPs probably contributing to the African versus non-African population differentiation in ZTGs. In contrast, the top Max F_ST_ gene values detected for *SLC39A4* when comparing any African and non-African population pair are caused by the extreme population differentiation of the L372V non-synonymous substitution (rs1871534), which presents a CADD Phred Score of 24.10.

### Zinc content of soil as an environmental selective pressure

**Figure 3:**
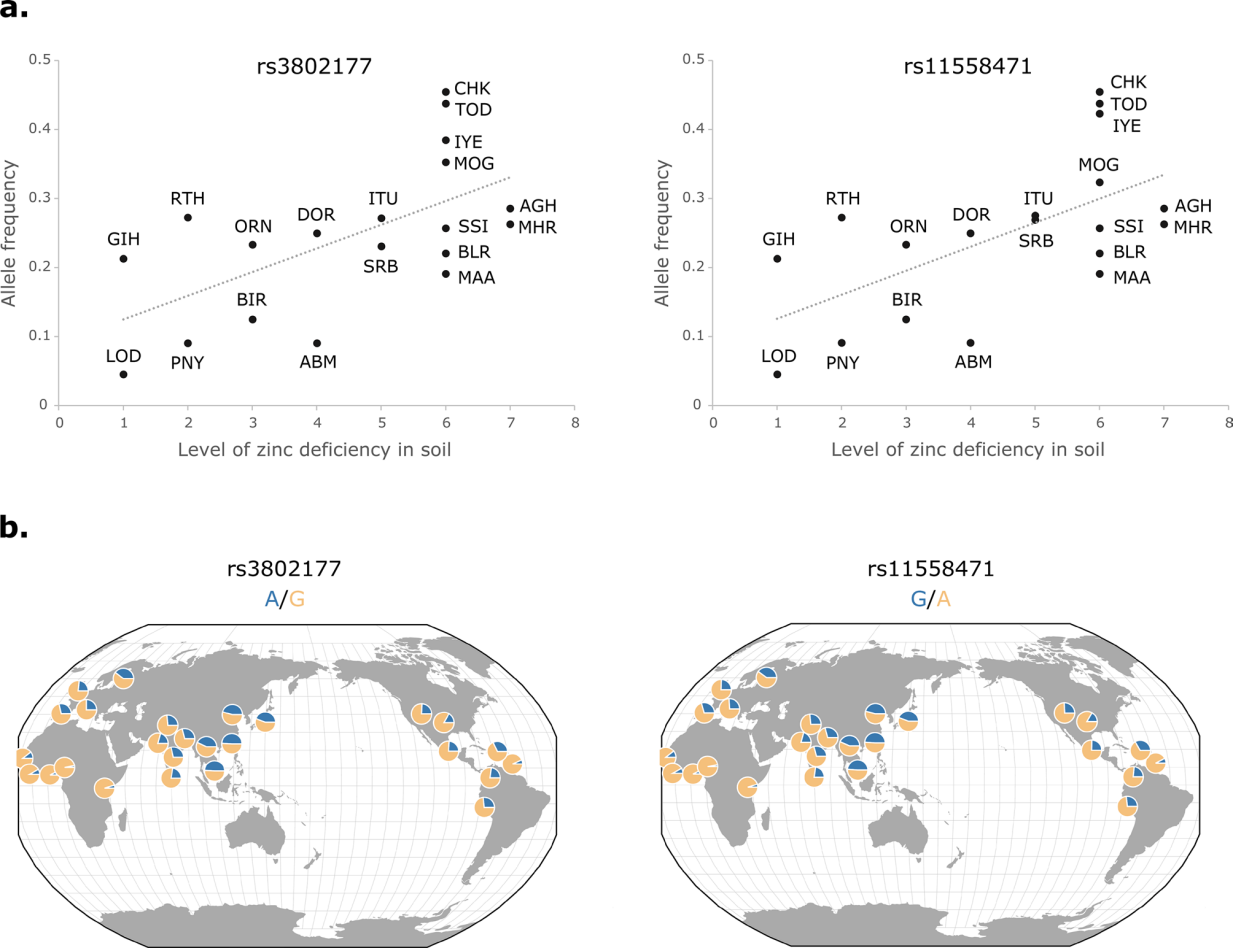
Allele frequencies for two UTR variants at the *SLC30A8* gene. (**a**) Frequencies for the derived A allele at rs3802177 (left) and the derived G allele at rs11558471 (right) in present-day populations from India plotted against zinc deficiency in soil in their assigned locations (for details, see “Methods” section). Number of individuals per population: GIH (101), LOD (11), RTH (11), PNY (11), ORN (15), BIR (12), DOR (12), ABM (11), ITU (127), SRB (13), CHK (11), TOD (16), IYE (13), MOG (17), SSI (35), BLR (34), MAA (34), AGH (14) and MHR (19). The full names for all populations and their corresponding allele frequencies are available in Supplementary Tables S3 and S15. (**b**) Worldwide allele frequency distribution for rs3802177 (left) and rs11558471 (right) in the populations of the 1000GP dataset. Blue and yellow indicate the minor and most common allele, respectively. Plots were obtained with the Geography of Genetic Variants Browser (version 0.4 (beta); https://popgen.uchicago.edu/ggv/).

As human zinc deficiency in India is well recognized^22^, we also used the South Asian dataset to investigate correlations between the zinc content of soil and the SNP genotype frequencies of ZTGs while considering the genetic structure of the analyzed South Asian population groups. Samβada’s multivariate analysis identified 66 genotypes at 59 SNPs from six ZTGs (*SLC30A3*, *SLC30A4*, *SLC30A8*, *SLS39A7*, *SLC39A9,* and *SLC39A11*) significantly correlated with soil zinc content (Supplementary Table S14). Up to 35 out of these 59 SNPs were eQTLs for *SLC39A9*, including a UTR5 variant (rs2168241) and an intronic SNP with a CADD Phred Score of 14.97 (rs17106979). One non-synonymous SNP with a CADD Phred Score of 12.79 was also detected for *SLC39A7* (rs1547387). Moreover, two of the SNPs with genotypes significantly correlating with soil zinc content (rs3802177 and rs11558471) have been previously associated with type 2 diabetes^45^, fasting plasma and blood glucose^46^, glycated hemoglobin levels^47^, proinsulin levels^48^, and body mass index^49^ and are located at the 3′ UTR of *SLC30A8*, which encodes an islet zinc transporter necessary for proper insulin secretion. In particular, when directly analyzing the correlation between allele frequencies and the zinc content of soil, the derived alleles of these two SNPs, which are in high linkage disequilibrium with each other (r^2^ > 0.85) and display almost identical frequencies worldwide, both show a significant positive correlation with zinc deficiency in soils (Spearman ρ = 0.60, p = 0.0061 for rs3802177; and ρ = 0.60, p = 0.0065 for rs11558471, respectively; Fig. 3).

## Discussion

In this study, we show that the complete set of ZTGs display higher genetic differentiation than expected in comparisons of African versus non-African populations. Several extremely differentiated ZTGs, namely *SLC30A9*, *SLC39A4* and *SLC39A5*, were found to consistently contribute to such coordinated changes of allele frequencies in most of these comparisons. Moreover, when aggregating other signatures of positive selection across all the ZTGs, significant signals of recent selection emerged in the patterns of linkage disequilibrium of ZTGs in distinct non-African populations of the 1000GP dataset, especially in those from South Asia. The main contributors to these signals were several ZTGs with unusual extended homozygous haplotypes (*SLC30A9*, *SLC30A10*, *SLC39A8* and *SLC39A11*). The same concerted adaptive response was replicated in our second dataset comprising only populations from South Asia, as ZTGs were unusually enriched for SNP variants associated with long haplotypes in several population groups, mostly non-tribal. Again, several ZTGs (*SLC30A9*, *SLC30A10*, *SLC39A10*, and *SLC39A11*) contributed to such coordinated signatures of recent positive selection. The signals detected by the iHS analysis were found to be more consistent across the different approaches applied to analyze enrichment for signatures of positive selection in the complete set of 24 ZTGs than those captured with XP-EHH, probably indicating the co-existence of different ongoing or incomplete sweeps across different ZTGs. Furthermore, in the South Asian dataset, we also detected genotype frequencies at six ZTGs (*SLC30A3*, *SLC30A4*, *SLC30A8*, *SLS39A7*, *SLC39A9,* and *SLC39A11*) significantly correlating with zinc deficiency levels in soil as a potential environmental selective pressure, while correcting for population structure. Therefore, a combination of selection signatures is probably contributing to the polygenic signal detected in South Asia: some subtle changes in genotype frequency correlating with zinc deficiency in soil and several strong signals of recent positive selection acting only on a few ZTGs.

In our analyses, we also identified several consistent outlier ZTGs for population differentiation, extended haplotype homozygosity, and an excess of rare variation in several populations from distinct geographical regions. These can be considered as putative candidates for local adaptation, probably resulting from hard selective sweeps. While several of these outlier genes appear to contribute to the global signatures of polygenic selection observed in South Asia, the very strong signals of positive selection identified in other geographical regions seem insufficient for the clear detection of unusual patterns of variation in the complete set of ZTGs in these regions. Some of these outlier ZTGs are well-recognized targets for positive selection in East Asia (*SLC30A9*)^8,33^, sub-Saharan Africa (*SLC39A4*)^36^ or elsewhere (*SLC39A11*)^8^, whereas other ZTGs are identified here for the first time as new putative candidates in several African populations (*SLC39A7*) as well as outside Africa and with strong signatures in South Asia (*SLC39A5*). Thus, ZTGs seem to have been a common target for strong positive selection in several geographical regions. In the previously reported cases, the strongest evidence for a classic selective sweep is found in *SLC30A9*, which has been attributed to a major favored haplotype nearly fixed in East Asia and at high intermediate frequencies in Europe and South Asia^8,29–32^. Moreover, a non-synonymous substitution (rs1047626) in *SLC30A9* and three linked nutriQTLs affecting zinc content in the liver have been proposed as putative adaptive variants behind such a strong signal of positive selection^8,33^. Here, we have identified several other highly differentiated linked eQTLs and SNPs with high CADD values associated with the same signal. Similarly, a clear selective sweep linked to another nutriQTL has already been described for *SLC39A8* in East Asia, the selection signal not been found in Africa or Europe^8,33^. Notably, our analysis provides evidence for extreme population differentiation in *SLC39A8* in South Asia (when comparing several South Asian groups with YRI) and reveals several eQTLs linked to the East Asian signature. In contrast with the *SLC30A9* and *SLC39A8* genes, *SLC39A4* presents an unusual pattern of positive selection with an extremely differentiated non-synonymous variant (rs1871534) recognized in several studies^34,35^, which displays high frequencies of the derived allele in sub-Saharan Africa but no other typical accompanying signals of selective sweeps because of a recombination hotspot in the region^36^. Accordingly, in our analyses, *SLC39A4* contributes to the African versus non-African genetic differentiation of ZTGs as a clear outlier gene together with *SLC30A9* only when using Max F_ST_.

Except for the two aforementioned non-synonymous variants of *SLC39A4* and *SLC30A9,* the other identified candidate SNPs are either eQTLs for different ZTGs, or genetic variants in conserved functional regions as inferred from their genome annotation, CADD values and other in silico predictions of potential functional relevance. In most cases, the joint putative adaptive response mediated by the complete set of ZTGs is thus expected to arise from coordinated changes in their regulation. Although usually less recognized as valid candidates, adaptive regulatory variants have great potential to mediate important adaptive responses in humans, as illustrated by the case of an upstream variant of the *TRPM8* cold receptor gene^50^. Moreover, since many of the putative adaptive variants in ZTGs are extremely differentiated between human populations from distinct geographical regions (mostly when comparing Africans with non-Africans), important differences in zinc homeostasis are expected across continents with potential consequences in different health traits.

Physiologically, it is interesting to observe that the ZTGs encoding the main intestinal zinc transporters in charge of nutritional zinc status through mechanisms of absorption (*SLC39A4*) and excretion (*SLC39A5*) are extremely differentiated between African and non-African populations. Whereas the strong differentiation of rs1871534 at *SLC39A4* has been suggested to arise from a selective event in sub-Saharan Africa^36^, we found that *SLC39A5* displays signals of positive selection outside Africa. Such an evolutionary pattern could suggest the existence of at least two parallel adaptive responses to ensure the appropriate intestinal zinc transport regulation, depending on zinc availability. Remarkably, the correlation analysis between genotype frequencies at ZTGs and zinc deficiency in soil in South Asia did not result in any significant hit for *SLC39A4,* the main transporter at the apical membrane of enterocytes responsible for the absorption of dietary zinc. On the contrary, all significant hits were for intracellular zinc transporters. Among these, *SLC30A3*, *SLC30A4* and *SLC30A8* accumulate zinc in intracellular compartments such as synaptic vesicles, lysosomes, and insulin vesicles, respectively, suggesting an adaptation that allows zinc to be stored in cells to maintain specific functions. Finally, two linked variants strongly correlated with environmental zinc levels have been associated with susceptibility to type 2 diabetes and other related phenotypes^45–48^. These are located at the 3’ UTR of the *SLC30A8* gene, which encodes the ZnT-8 transporter involved in the regulation of insulin secretion in pancreatic β cells.

The increasing availability of sequencing data from non-reference populations and additional ethnic minority groups is providing new insights into the evolutionary history of human populations. Here, the inclusion and analysis of the pilot phase of the GenomeAsia 100 K Project^42^, together with other publicly available sequencing datasets covering South Asia, proved pivotal for the determination of unusual patterns of adaptation in the complete set of ZTGs in a geographical region where zinc deficiency is a recognized public health problem. We note, however, that the use of currently available data on zinc deficiency in the environment is complex, especially when comparing large areas across the globe, as they are not usually standardized. Furthermore, zinc deficiency may have also changed along human history and current data can only be used as a proxy for zinc levels in the past. Moreover, within India, dietary practices are likely to differ by culture, region and/or socioeconomic status, with a varying proportion of plant- versus animal-based foods, which may further confound any association between local zinc levels in soil and genetic variation in ZTGs.

Zinc deficiency in soils has been inferred as the main driving force behind the signals of positive selection in South Asia described in this study. However, we recognize that other selection pressures could also be involved. Indeed, zinc homeostasis clearly influences human health status, especially the immune response^16,17^, which may have been relevant for our survival and response to local pathogens. Moreover, although the complete set of ZTGs were analyzed together, because they code for different zinc transporters, some of the transporters (i.e., ZnT10, ZIP4, ZIP8 or ZIP14) are known to transport other elements such as Zn^2+^, Fe^2+^, Mn^2+^, or Cd^2+^^14^. Thus, for such ZTGs, local selective pressures related to the availability of other micronutrients cannot be discarded. Finally, as zinc transporters are related to additional important physiological functions and signaling processes besides zinc transport (i.e., insulin secretion, neuronal signaling, and regulation of the immune response, among others), other alternative adaptive hypotheses could be suggested. Only a detailed functional characterization for each putative adaptive variant and subjacent phenotype will allow a comprehensive understanding of the molecular phenotypes and putative selection pressures behind the selection signatures in the complete set of ZTGs.

## Methods

### Whole-genome sequencing data

Sequencing data were extracted from phase 3 of the 1000 Genomes Project^41^ (1000 GP; May 2013 release, version 3.4). All offspring trios, as well as the Americans of African Ancestry in South-West USA (ASW) and African Caribbeans in Barbados (ACB) populations, were excluded. Only the biallelic variants from the VCF files were kept for analysis. The resulting 1000GP dataset comprised 80,855,722 SNPs in 2,328 unrelated individuals from 24 populations, which can be grouped in five main geographical regions (for details, see Supplementary Table S1). We also compiled sequencing data from the Pilot phase of the GenomeAsia 100 K Project^42^ and two other publicly available datasets: the Singapore Sequencing Indian Project (SSIP)^51^ and the Simons Genome Diversity Project (SGDP)^52^. From these, only individuals belonging to the South Asian region (i.e., from Pakistan, India, Nepal, Bangladesh, and Sri Lanka) were considered for analysis. Sequencing data from the five South Asian populations (BEB, GIH, ITU, PJL, and STU) and one reference population from each of the remaining geographical regions (CEU, CHB, MXL, and YRI) in the 1000 GP (for details, see Supplementary Table S1, S2) were extracted and merged with the compiled South Asian sequences to obtain an extended South Asian dataset with external populations. For that, we first applied a standard quality control protocol in the four original datasets using PLINK 1.9^53^ to filter out individuals with more than 10% of missing calls and SNPs missing in more than 5% of individuals. Related samples in each dataset were then removed using the KING-robust estimator^54^ available in PLINK 2.0, with a cutoff of 0.0884 to screen for second-degree relatives. Finally, all variants with a Minor Allele Frequency (MAF) below 2% in the new merged dataset were removed using PLINK 1.9. The resulting extended South Asian dataset comprised 1,517 individuals and 5,951,093 SNPs (Supplementary Table S2).

### Genetic structure in the South Asian dataset

Genetic structure was explored employing Principal Component Analysis (PCA) and ADMIXTURE. For that, we first pruned the South Asian dataset for linkage disequilibrium with PLINK 1.9 by removing one SNP of each pair of SNPs in a window of 50 SNPs when r^2^ was greater than 0.5 and shifting the window 5 SNPs forward each time. After pruning, the dataset included 957,919 SNPs and 1517 samples from 74 populations (Supplementary Table S2). PCA was executed using the SmartPCA program in the EIGENSOFT 6.0.1 package^55^ and visualized using an in-house R script. As for ADMIXTURE^56^, we performed five runs with different random seeds for 2 to 11 ancestral components (K) and conducted a cross-validation (CV) procedure to measure the fittest K value, visualizing it with pong^57^. After inspection of these initial PCA and ADMIXTURE analyses (Supplementary Fig. S1), we formed eight homogenous groups of South Asian populations with sample sizes > 30, considering geographical, language and social criteria, to be analyzed together with one population representative of four other geographical areas (YRI for Africa, CEU for Europe, CHB for East Asia and MXL for America). The resulting South Asian dataset consisted of 1,353 samples and 5,953,446 SNPs (for details, see Supplementary Table S3). After pruning, PCA and ADMIXTURE analyses were carried out as before to visualize the genetic structure of the South Asian groups used for the selection analyses (Fig. 1, Supplementary Fig. S2).

### Zinc Transporter Genes (ZTGs) and reference genes

To evaluate whether the complete set of ZTGs deviates from neutral expectations, we first compiled genomic data on the 24 Zinc Transporter Genes (ZTGs) known in humans and then looked for genome-wide reference genes with similar genomic characteristics to each of the ZTGs. For that, we extracted the gene coordinates, GC content, and coding sequence length for the longest coding transcript of all 20,314 human autosomal protein genes available on build 37 (hg19) using the BioMart interface from the Ensembl genome browser. Gene recombination rates were computed using the weighted recombination rates for the corresponding overlapping genomic regions as available in the UCSC database. Reference genes selected for each ZTG were those that differed less than 20% in length, GC content, and recombination rate. Overall, 5146 genes matched these criteria for at least one ZTG (Supplementary Table S4). Sequencing data for all ZTGs and their corresponding reference genes were then extracted from the two compiled datasets obtaining two independent working datasets to be used for the selection analyses: the 1000GP dataset with 2,328 worldwide samples and the South Asian dataset with 1353 South Asian and reference population samples (Supplementary Tables S1, S3, respectively). In each case, the statistical significance of the whole set of 24 ZTGs when testing for evidence of positive selection was evaluated by generating 10,000 random subsets of 24 reference genes with similar characteristics to each of the 24 ZTGs from the 5146 matched genes pool compiled (for details, see Supplementary Fig. S3).

### Population differentiation

To explore for unusual patterns of population differentiation, SNP F_ST_ values for all pairs of populations in the 1000GP dataset, as well as between all South Asian population groups and the YRI population, were calculated using the Weir and Cockerham estimator^58^ as available in VCFtools^59^. For each gene, we retrieved the highest F_ST_ value (Max F_ST_) and the weighted average F_ST_ value (WA F_ST_) computed as in White et al.^7^.

To evaluate whether the levels of population differentiation for the complete set of ZTGs differed from genome-wide expectations, we implemented two approaches. On the one hand, we calculated the mean WA F_ST_ for all the ZTGs and for every subset of 24 randomly matched genes, and computed the corresponding rank value of the ZTGs, which was considered as a p-value; this procedure was also followed with the Max F_ST_ values. On the other hand, we also used a permutation test to analyze whether ZTGs present a greater proportion of highly differentiated SNPs than randomly matched genome-wide genes. For that, we considered the 99th percentile of all the SNP F_ST_ values obtained in each resampling and population comparison, computed the proportion of ZTG SNPs above such an empirical cutoff, and used as the p-value the fraction of times that in the 10,000 resamplings the proportion of highly differentiated SNP in the ZTGs was lower than that of the predefined cutoff (for details, see Supplementary Fig. S3).

### Exploring signals of positive selection

Signals of recent positive selection were investigated using two methods based on the extension of the haplotype homozygosity: the integrated Haplotype Score (iHS)^30,43^ and the cross-population Extended Haplotype Homozygosity (XP-EHH)^30,43^. Since both statistics explore for unusual long-range haplotypes and recombination rapidly breaks down this signature on the pattern of linkage disequilibrium, they are powerful tests to interrogate selection events dating < 30 kya: iHS when the selected allele is polymorphic at intermediate frequencies and XP-EHH for selected variants at high frequency or nearly fixed in the tested population^30^. For every population in each dataset and for each variant for which ancestral allele information was available, we calculated the standardized iHS values using Selscan with its default parameters^60^ and used the iHS absolute values, from now on referred to as iHS values, to calculate the corresponding average (and maximum) gene values of the statistic. We also used Selscan’s default parameters to compute XP-EHH SNP values pairwise across five populations, each representing a main geographical region in the 1000GP dataset (i.e., YRI from Africa, CEU from Europe, CHB from East-Asia, GIH from South-Asia, and MXL from America), as well as for every South Asian population group using CHB as the reference population. The sign of the XP-EHH values was kept to analyze the positive and negative values separately to be able to infer the direction of the selection signal in each corresponding population.

As in the F_ST_ analysis above, for every gene in our analysis (ZTGs and randomly matched genes) we computed the highest iHS and XP-EHH values per gene (Max iHS and Max XP-EHH, respectively) and the average iHS and XP-EHH values per gene (Avg iHS and Avg XP-EHH, respectively). Similarly, we also performed a rank test to determine whether the obtained iHS and XP-EHH mean values for the whole set of ZTGs differed from genome-wide expectations and a permutation test to check whether ZTGs had a greater than expected proportion of SNPs with extreme iHS and XP-EHH values (for procedures, see Supplementary Fig. S3). For the rank test, in the event of obtaining no iHS or XP-EHH value computed for a particular ZTG and population, that specific ZTG and its matching genes were excluded from the analysis of that population. It should also be noted that when a strong signal of positive selection is detected in the reference or tested population, no XP-EHH value is obtained for the other population.

Deviations in the site frequency spectrum were investigated using the Tajima’s D neutrality test^61^. For each population and gene in the 1000GP dataset, an individual VCF file was generated. Tajima’s D values were then computed for each gene individually using VCFtools. Each gene set was then assigned the mean value of the corresponding individual gene scores. The significance of Tajima’s D value for the complete set of ZTGs was evaluated by comparing it to genome-wide expectations through a rank test using 10,000 resamplings of randomly matched genes as described before (for the procedure, see Supplementary Fig. S3).

Finally, we also used the SUMSTAT statistic^44^ to test whether the sum of the maximum gene scores of each statistic (i.e., Max F_ST_, Max XP-EHH and Max iHS) in the whole set of ZTGs was higher than expected considering the corresponding values of 10,000 sets of 24 random gene sets matched for gene length, recombination, and GC content, while controlling for their SNP density. For that, all genes were first assigned to 13 bins according to their number of SNPs to control for SNP density and a standardized maximum statistic score was measured for each gene following the strategy of Daub et al.^40^. Gene standardized scores in each set of 24 genes were then summed, and the significance of the SUMSTAT test for the whole set of 24 ZTGs was assessed by comparing it to that of the 10,000 resamplings of randomly matched genes.

### Identification of individual candidate genes and variants among ZTGs

Per each population comparison, we considered ZTGs to be highly differentiated if they had WA F_ST_ (or Max F_ST_) values above the 99th percentile in the global distribution of WA F_ST_ (or Max F_ST_) values obtained from the 5146 reference genes independently for the same population comparison. Similarly, to identify which individual ZTGs contributed most to the potential signals of selection detected for the complete set of 24 ZTGs, we ranked the mean gene value obtained for each statistic (iHS, XP-EHH, and Tajima’s D) and population across the 5146 genome-wide reference genes and identified as putative outliers those ZTGs within the top 1% of the corresponding statistic (for the procedure, see Supplementary Fig. S4).

To recognize which SNPs contributed most to the population differentiation of ZTGs (F_ST_) and to the detected iHS and XP-EHH signals, we looked for SNPs in ZTGs whose statistical score was above the 1% cutoff of the SNP scores of 24 randomly matched genes in at least 95% of the 10,000 resampling sets (for details, see Supplementary Fig. S4). Outlier SNPs were subsequently annotated using the ANNOVAR software^62^ to obtain gene-based annotations (such as gene variant location and non-synonymous changes) and several in silico predictions of their potential functional relevance including the CADD score^63^, the Eigen score^64^ and the FitCons score^65^. We considered as functionally relevant those candidate SNPs with either a CADD Phred Score over 10, an Eigen Score > 0, an Eigen PC Score > 0, or a FitCons p-value < 0.003. We also extracted and annotated as functionally relevant all the available associated information for these candidate SNPs in the GTEx Portal Dataset v7^66^ and the GWAS Catalog v1.0^67^.

Finally, we used the Samβada software^68^ to explore the South Asian dataset for correlations between SNP genotype frequencies at ZTGs and zinc content in soil, while correcting for population structure using a multivariate model. For that, we first assigned a unique geographical location to each Indian population of the South Asian dataset, according to available information in the GenomeAsia 100 K Project^42^ and complementary sources, and inferred a percentage of zinc deficiency in soil to each location from data uniformly quantified and available for different agro-ecological regions of India^69^ (Supplementary Table S3). The first and the second Principal Components were used to correct the correlation analysis for population structure, as recommended^68^. The G and Wald scores were obtained for all genotypes analyzed to generate the corresponding p-values that were later corrected for multiple testing using the Benjamini–Hochberg method. Spearman correlations between the allele frequencies of candidate SNPs of interest and the percentage of soil samples deficient in zinc in their assigned location were also computed, considering only populations with sample sizes of more than 10 individuals and according to the zinc deficiency values provided in Supplementary Table S3. Allele frequency plots and maps were obtained with the Geography of Genetic Variants Browser (version 0.4 (beta); https://popgen.uchicago.edu/ggv/)^70^.

### Ethics declaration

The study was approved by the author’s institutional review board (CEIm—Parc de Salut MAR, reference number 2019/8916/I).

## Supplementary Information

Supplementary Information.

Supplementary Tables.

## Data Availability

All sequencing data analyzed in this study are published publicly available data from the 1000 Genomes Project (https://www.internationalgenome.org/) and the GenomeAsia 100 K Project. GenomeAsia 100 k individual VCF files are available through the European Genome Archive EGA under accession # EGAS00001002921.
