# Author Correction: Understanding signatures of positive natural selection in human zinc transporter genes

**DOI:** 10.1038/s41598-022-09566-2

**Published:** 2022-03-30

**Authors:** Ana Roca-Umbert, Rocio Caro-Consuegra, Diego Londono-Correa, Gabriel Felipe Rodriguez-Lozano, Ruben Vicente, Elena Bosch

**Affiliations:** 1 Institut de Biologia Evolutiva (UPF-CSIC), Departament de Medicina i Ciències de la Vida, Universitat Pompeu Fabra, Parc de Recerca Biomèdica de Barcelona, 08003 Barcelona, Spain; 2 Laboratory of Molecular Physiology, Universitat Pompeu Fabra, Parc de Recerca Biomèdica de Barcelona, 08003 Barcelona, Spain; 3 Centro de Investigación Biomédica en Red de Salud Mental (CIBERSAM), 43206 Reus, Spain

Correction to: *Scientific Reports* 10.1038/s41598-022-08439-y, published online 12 March 2022

The original version of this Article contained an error in Fig. 1(A) where some of the labels in the key were incorrect. As a result, the Indian population names for T-DR, T-AA, T-TB, nT-DR, and nt-IE were incorrectly given as A-DR, A-AA, A-TB, C-DR, and C-IE respectively.

**Figure 1:**
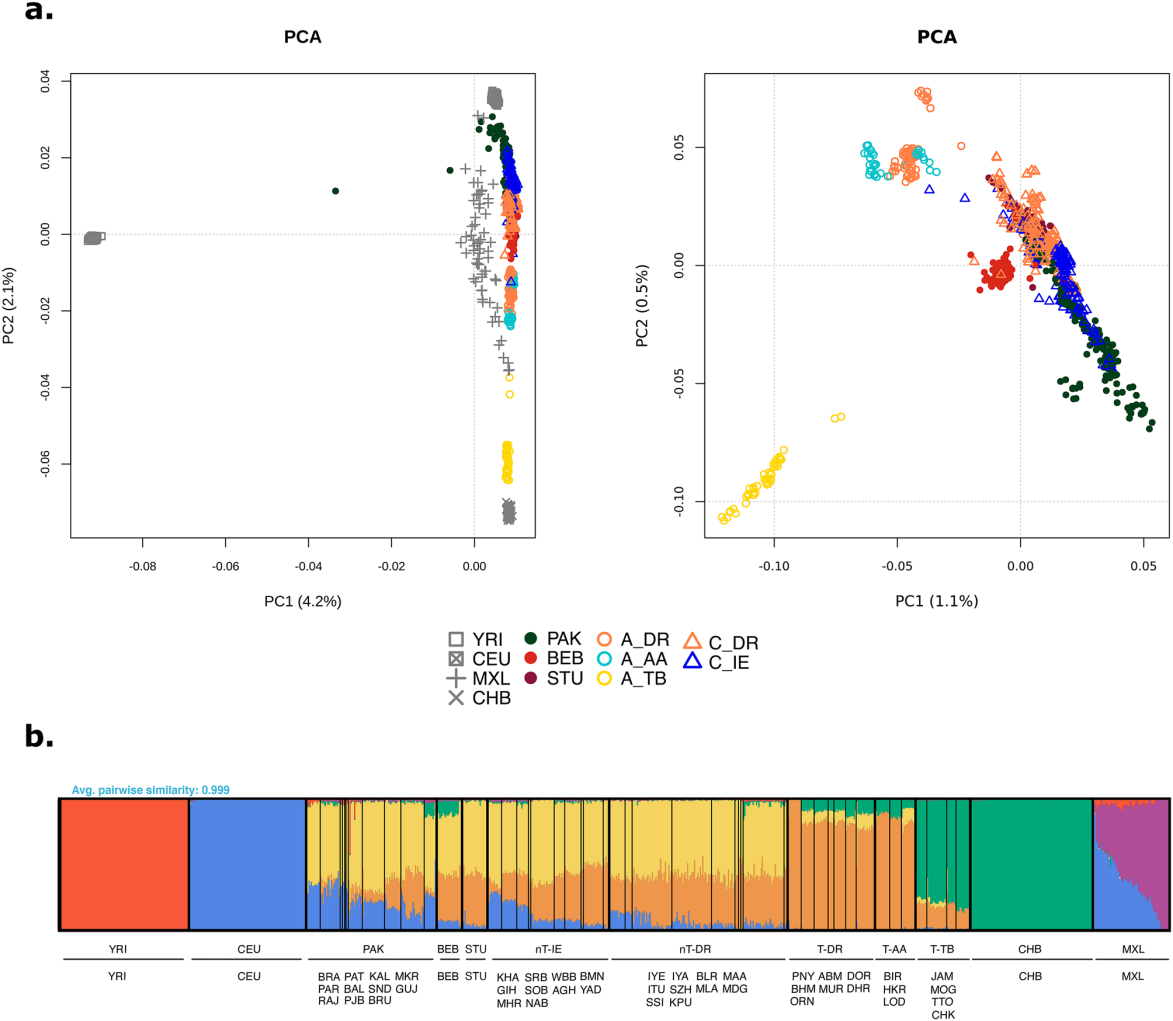
Population structure analysis of the South Asian dataset. (**a**) Principal Component Analysis (PCA) of the curated dataset with (left) and without (right) external reference populations. (**b**) ADMIXTURE analysis of the curated dataset, K = 6 (CV error = 0.4274). For populations from the 1000 GP belonging to the South Asian region, a subset of 15 samples is represented. Population group abbreviations: *T-AA* tribal populations speaking Austroasiatic languages, *T-DR* Dravidian-speaking tribal populations, *T-TB* Tibeto-Burman-speaking tribal populations, *nT-DR* non-tribal populations speaking Dravidian languages, *nT-IE* non-tribal populations speaking Indo-European languages, *PAK* Pakistan, *BEB* Bangladesh, *STU* Sri Lanka, *YRI* Yoruba in Ibadan, Nigeria, Africa, *CEU* Utah residents with Northern and Western European ancestry, *CHB* Han Chinese in Beijing, China, *MXL* individuals with Mexican ancestry from Los Angeles, California. The full names for all populations within groups are available in Supplementary Table S3.

The original Fig. 1 and accompanying legend appear below.

The original Article has been corrected.

