# Supplementary material for: Understanding signatures of positive natural selection in human zinc transporter genes
Source: Sci Rep. 2022 Mar 12;12:4320. doi: 10.1038/s41598-022-08439-y (PMC8918337; doi:10.1038/s41598-022-08439-y)
Supplement: Supplementary file 1 — Supplementary Information. [file 41598_2022_8439_MOESM1_ESM.pdf]

Understanding signatures of positive natural selection in human zinc transporter genes

Ana Roca-Umbert[1], Rocio Caro-Consuegra[1], Diego Londono-Correa[1], Gabriel Felipe Rodriguez-Lozano[1], Ruben Vicente[2], Elena Bosch*[1,3]

[1]Institut de Biologia Evolutiva (UPF-CSIC), Departament de Ciències Experimentals i de la Salut, Universitat Pompeu Fabra, Parc de Recerca Biomèdica de Barcelona, 08003 Barcelona, Spain

[2]Laboratory of Molecular Physiology, Universitat Pompeu Fabra, Parc de Recerca Biomèdica de Barcelona, 08003 Barcelona, Spain

[3]Centro de Investigación Biomédica en Red de Salud Mental (CIBERSAM), 43206 Reus, Spain

*Corresponding author: elena.bosch@upf.edu

Supplementary Information

- **Supplementary Figures S1-S8**
- **Supplementary Note 1**
- **Supplementary Figures S1-S14 (excel file)**

a

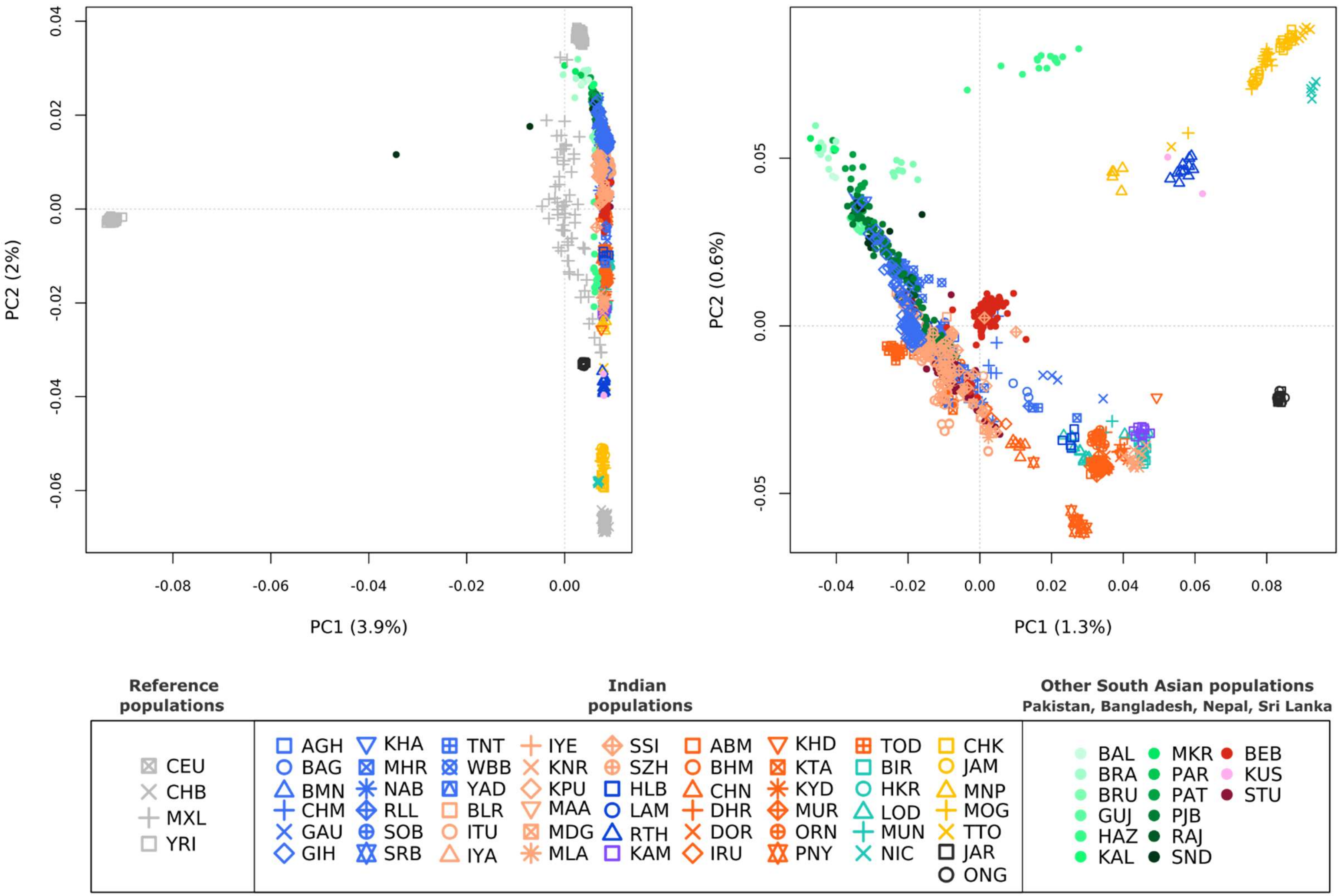
PC1 (3.9%)
PC2 (2%)
-0.08
-0.06
-0.04
-0.02
0.00
0.04
0.02
0.00
-0.02
-0.04
-0.06
PC1 (1.3%)
PC2 (0.6%)
-0.04
-0.02
0.00
0.02
0.04
0.06
0.08
0.05
0.00
-0.05
Reference populations
CEU
CHB
MXL
YRI
Indian populations
AGH
BAG
BMN
CHM
GAU
GIH
KHA
MHR
NAB
RLL
SOB
SRB
TNT
WBB
YAD
BLR
ITU
IYA
IYE
KNR
KPU
MAA
MDG
MLA
SSI
SZH
HLB
LAM
RTH
KAM
ABM
BHM
CHN
DHR
DOR
IRU
KHD
KTA
KYD
MUR
ORN
PNY
TOD
BIR
HKR
LOD
MUN
NIC
CHK
JAM
MNP
MOG
TTO
JAR
ONG
Other South Asian populations
Pakistan, Bangladesh, Nepal, Sri Lanka
BAL
BRA
BRU
GUJ
HAZ
KAL
MKR
PAR
PAT
PJB
RAJ
SND
BEB
KUS
STU

b

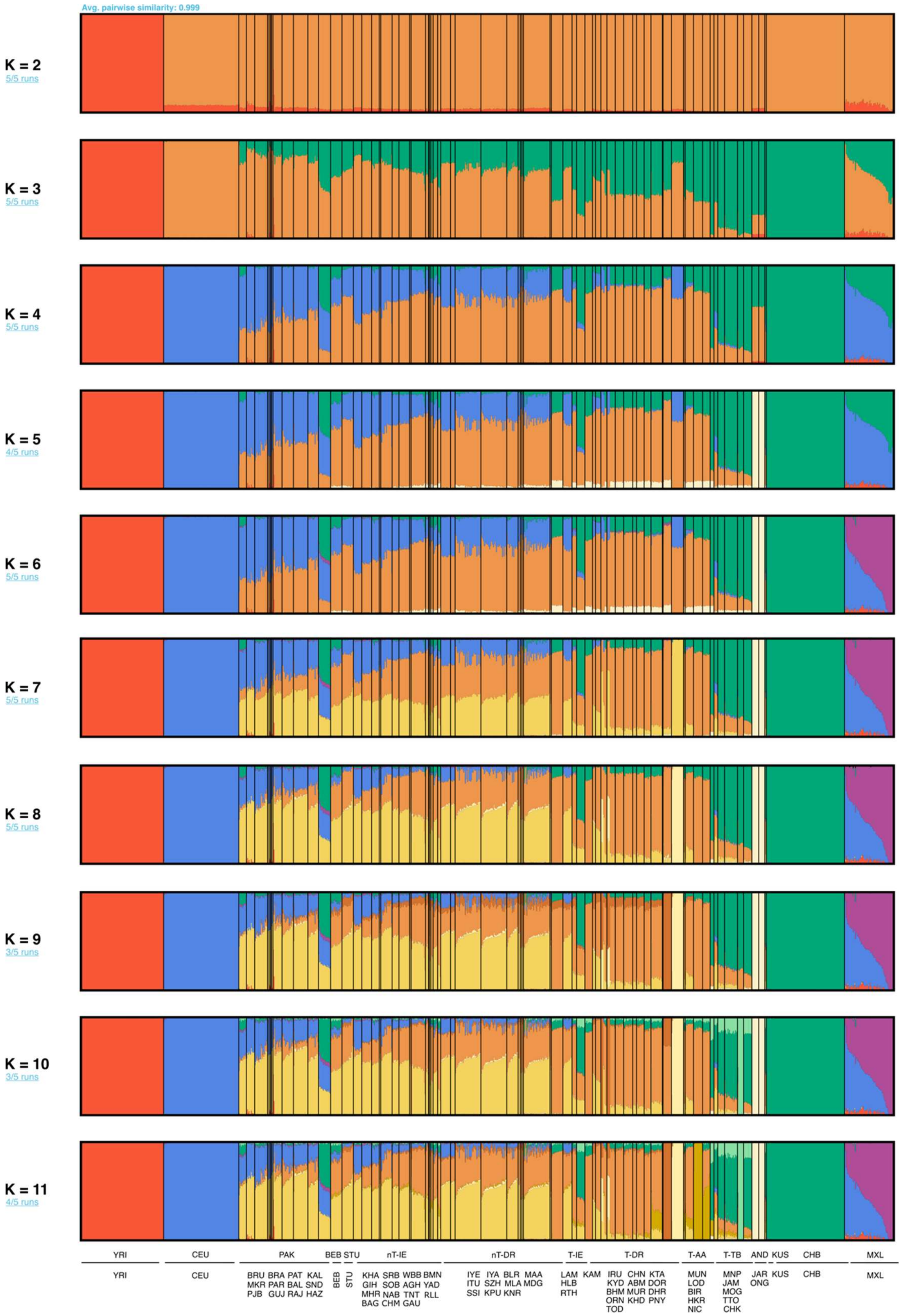
Avg. pairwise similarity: 0.999
K = 2
5/5 runs
K = 3
5/5 runs
K = 4
5/5 runs
K = 5
4/5 runs
K = 6
5/5 runs
K = 7
5/5 runs
K = 8
5/5 runs
K = 9
3/5 runs
K = 10
3/5 runs
K = 11
4/5 runs
YRI
CEU
PAK
BEB STU
nT-IE
nT-DR
T-IE
T-DR
T-AA
T-TB
AND KUS
CHB
MXL
YRI
CEU
BRU BRA PAT KAL
MKR PAR BAL SND
PJB GUJ RAJ HAZ
BEB
STU
KHA SRB WBB BMN
GIH SOB AGH YAD
MHR NAB TNT RLL
BAG CHM GAU
IYE IYA BLR MAA
ITU SZH MLA MDG
SSI KPU KNR
LAM KAM
HLB
RTH
IRU CHN KTA
KYD ABM DOR
BHM MUR DHR
ORN KHD PNY
TOD
MUN
LOD
BIR
HKR
NIC
MNP
JAM
MOG
TTO
CHK
JAR KUS
ONG
CHB
MXL

c

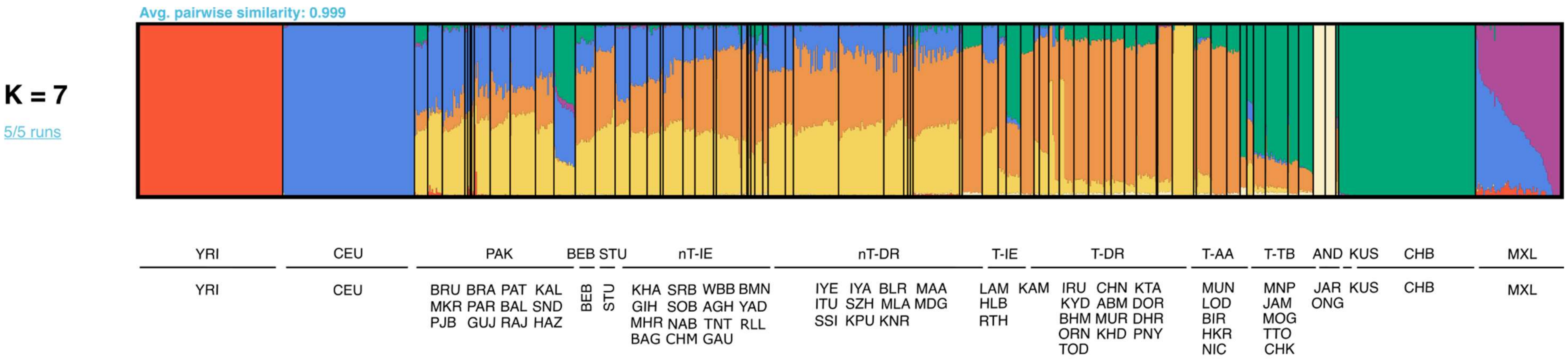

Figure S1. Population structure of the South Asian dataset before curation. (a). Plots of the first two Principal Components (PC) of the Principal Component Analysis (PCA). On the left all populations included in the South Asian dataset analysed with external reference populations (in grey) and on the right all the South Asian dataset analysed without external reference populations. (b). ADMIXTURE plot. According to CV error, K=7 is the best population structure representation for this dataset. (c) Enlarged ADMIXTURE analysis plot for K=7 detailing all the South Asian groups and populations included. Abbreviations for samples in each of these defined groups are described in Supplementary Table S2. Population group abbreviations: YRI (Yoruba in Ibadan, Nigeria, Africa), CEU (Utah residents with Northern and Western European ancestry), PAK (populations from Pakistan), BEB (individuals from Bangladesh), STU (individuals from Sri Lanka), NT-IE (non-tribal populations speaking Indo-European languages, NT-DR (non-tribal populations speaking Dravidian languages), A_IE (Indo-European-speaking tribal populations, T-DR (Dravidian-speaking tribal populations), T-AA (tribal populations speaking Austroasiatic languages), T-TB (Tibeto-Burman-speaking tribal populations), AND (populations from the Andaman Islands), KUS (individuals from Nepal), CHB (Han Chinese in Beijing, China), MXL (individuals with Mexican ancestry from Los Angeles, California).

a

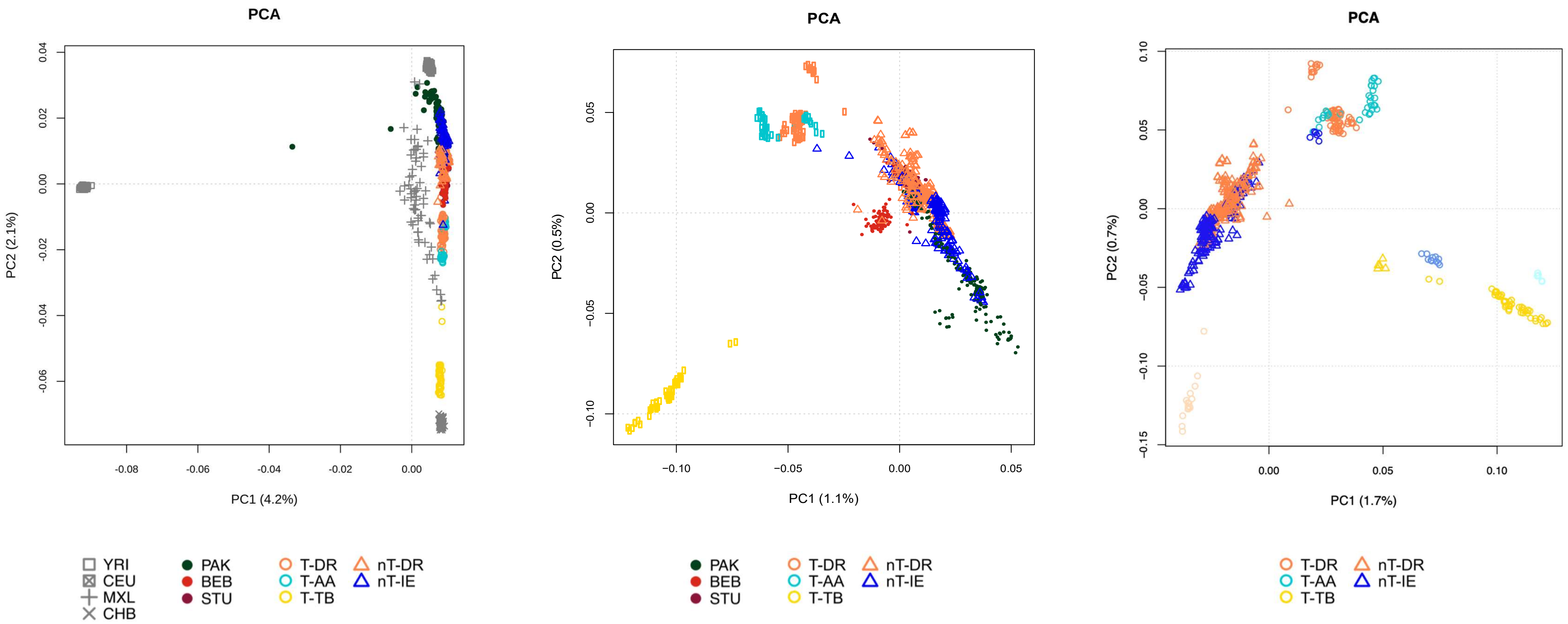
PCA
PC2 (2.1%)
PC1 (4.2%)
0.04
0.02
0.00
-0.02
-0.04
-0.06
-0.08
-0.06
-0.04
-0.02
0.00
PCA
PC2 (0.5%)
PC1 (1.1%)
0.05
0.00
−0.05
−0.10
−0.10
−0.05
0.00
0.05
PCA
PC2 (0.7%)
PC1 (1.7%)
0.10
0.05
0.00
−0.05
−0.10
−0.15
0.00
0.05
0.10
YRI
CEU
MXL
CHB
PAK
BEB
STU
T-DR
T-AA
T-TB
nT-DR
nT-IE

b

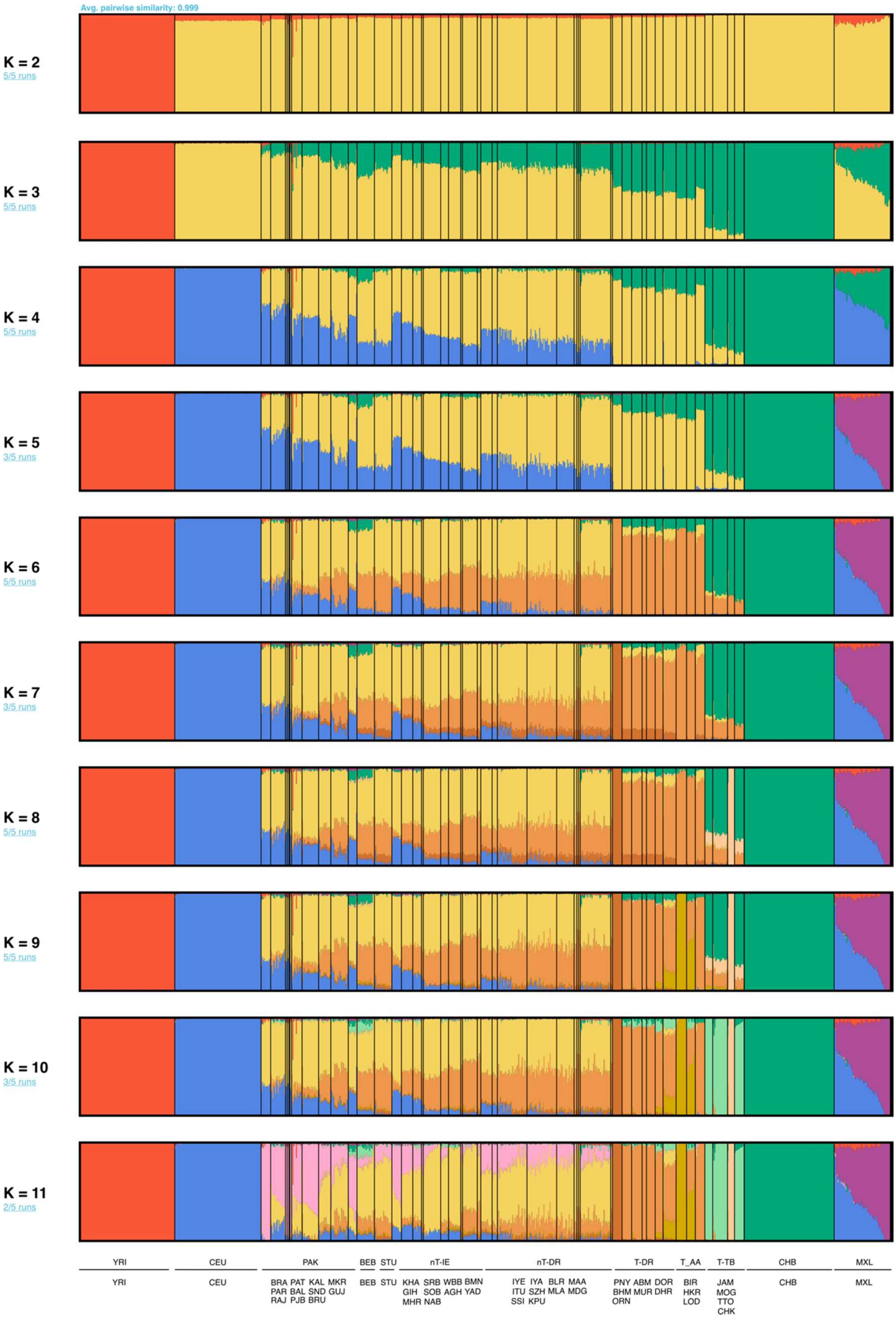
Avg. pairwise similarity: 0.999
K = 2
5/5 runs
K = 3
5/5 runs
K = 4
5/5 runs
K = 5
3/5 runs
K = 6
5/5 runs
K = 7
3/5 runs
K = 8
5/5 runs
K = 9
5/5 runs
K = 10
3/5 runs
K = 11
2/5 runs
YRI
CEU
PAK
BEB
STU
nT-IE
nT-DR
T-DR
T_AA
T-TB
CHB
MXL
YRI
CEU
BRA PAT KAL MKR
PAR BAL SND GUJ
RAJ PJB BRU
BEB
STU
KHA SRB WBB BMN
GIH SOB AGH YAD
MHR NAB
IYE IYA BLR MAA
ITU SZH MLA MDG
SSI KPU
PNY ABM DOR
BHM MUR DHR
ORN
BIR
HKR
LOD
JAM
MOG
TTO
CHK
CHB
MXL

Figure S2. Population structure of the South Asian dataset after curation. (a). Plots of the first two Principal Components (PC) of the PCA Analysis. Left: entire South Asian curated dataset including external reference populations (12 population groups); Middle: South Asian dataset without external reference populations (8 groups); Right: Indian populations groups (5 population groups). The individual populations included in each of these defined population groups are described in Supplementary Table S.3. (b). ADMIXTURE analysis representation plots for 2 to 11 ancestral components (K). According to CV error, K=7 is the best population structure representation for this dataset. Population groups abbreviations: YRI (Yoruba in Ibadan, Nigeria, Africa), CEU (Utah residents with Northern and Western European ancestry), PAK (populations from Pakistan), BEB (individuals from Bangladesh), STU (individuals from Sri Lanka), NT-IE (non-tribal populations speaking Indo-European languages, NT-DR (non-tribal populations speaking Dravidian languages), T-DR (Dravidian-speaking tribal populations), T-AA (Austroasiatic-speaking tribal populations), T-TB (Tibeto-Burman-speaking tribal populations), CHB (Han Chinese in Beijing, China), MXL (individuals with Mexican ancestry from Los Angeles, California).

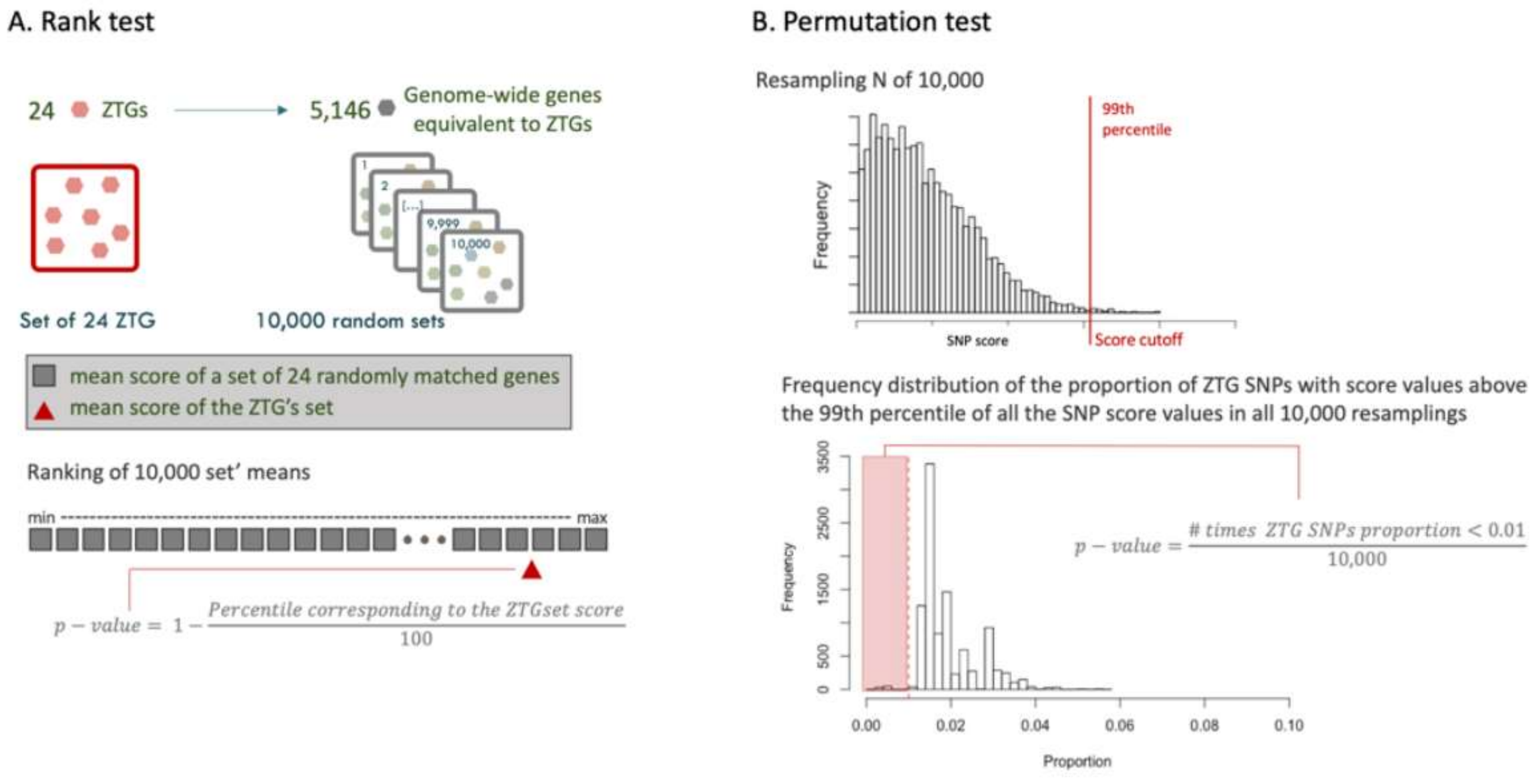

Figure S3. Methods to detect unusual patterns of population differentiation and signatures of positive selection in the whole set of 24 ZTGs. (A). Rank test to compare ZTGs with genome-wide matched genes**.** For each ZTG and 5,146 genome-wide equivalent genes, we first calculated F_{ST}, iHS and XP-EHH values per SNP and then obtained the maximum and average gene score of each statistic (except for F_{ST} where the weighted average F_{ST} value was used). Subsequently, the mean score of the 24 ZTGs set was ranked with that of 10,000 sets of 24 randomly matched genes to obtain the corresponding p-value in each population comparison (F_{ST} and XP-EHH) or population (iHS). (B). Permutation test to compare the proportion of outlier SNPs for a given statistic in ZTGs with that of genome-wide matched genes. For each set of 24 randomly matched genes and population comparison (or population), we considered the 99th percentile of all the SNP score values (top 1%) in each resampling and computed the proportion of ZTG SNPs above such empirical cutoff. Subsequently, we used as p-value the fraction of times that the proportion of ZTG SNPs in the 10,000 resamplings was lower than the 0.01 cutoff.

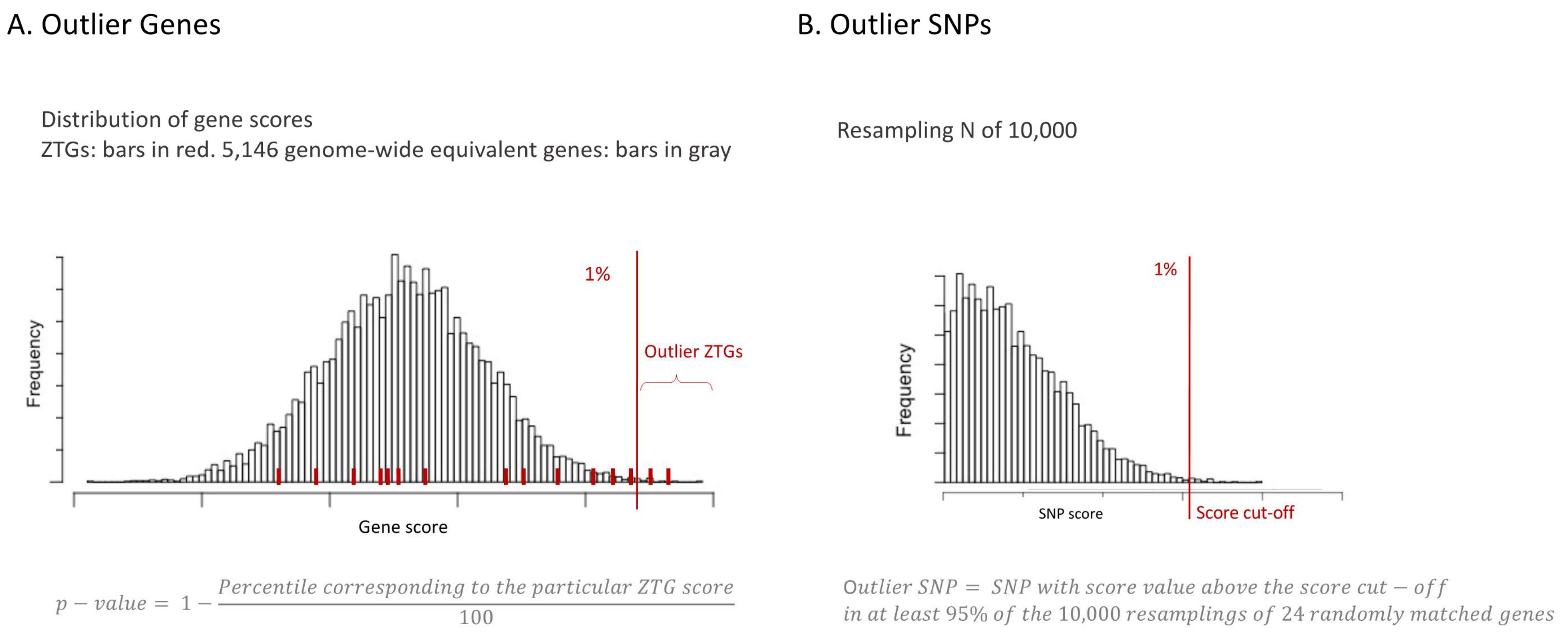

Figure S4. Contribution of individual genes and SNPs to unusual patterns of population differentiation and signatures of positive selection in ZTGs. (A) Identification of outlier ZTGs. For each statistic, we considered as outliers those ZTGs above the top 1% of a genome-wide distribution obtained from 5,146 similar genes. For each population comparison (F_{ST} and XP-EHH) or population (iHS), rank p-values per each individual ZTG were obtained from this genome-wide distribution as shown. (B) Identification of outlier ZTG SNPs. For each statistic and population comparison (or population), outlier SNPs in ZTGs were identified as those presenting score values above the corresponding top 1% SNP values in more than 9,500 resamplings of 24 genome-wide random matched genes.

a.

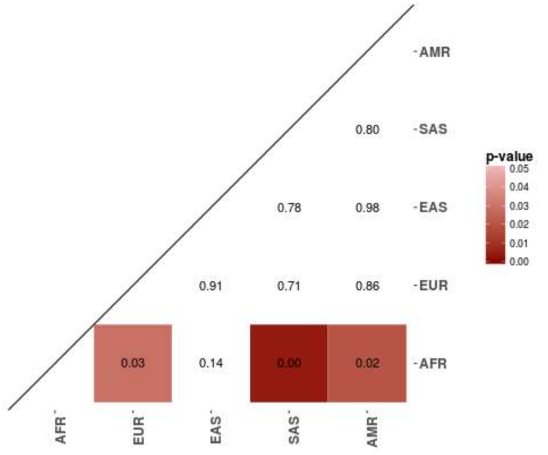

b.

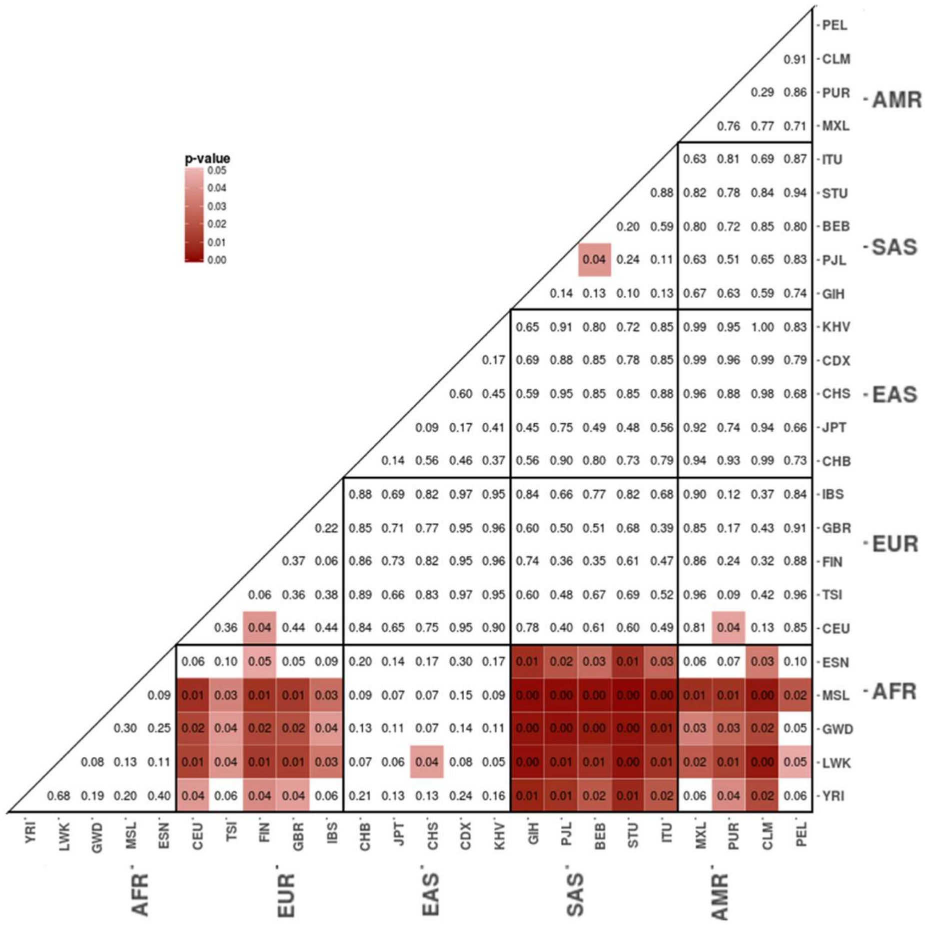

Figure S5. ZTGs present higher differentiation than randomly matched genes in African versus non-African population comparisons when comparing the average Max F_{ST} values. (a) For each F_{ST} comparison between geographical regions in the 1000GP dataset, p-values of the corresponding rank test when comparing the average Max F_{ST} value of the whole set of 24 ZTGs with that of 10,000 resamplings of 24 genome-wide matched genes. (b) For each F_{ST} population comparison in the 1000GP dataset, p-values of the corresponding rank test when comparing the average Max F_{ST} value of the whole set of 24 ZTGs with that of 10,000 resamplings of 24 genome-wide matched genes.

a.

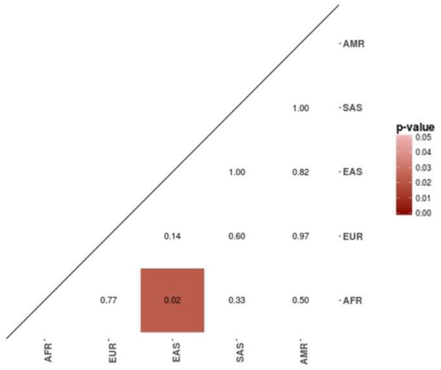

b.

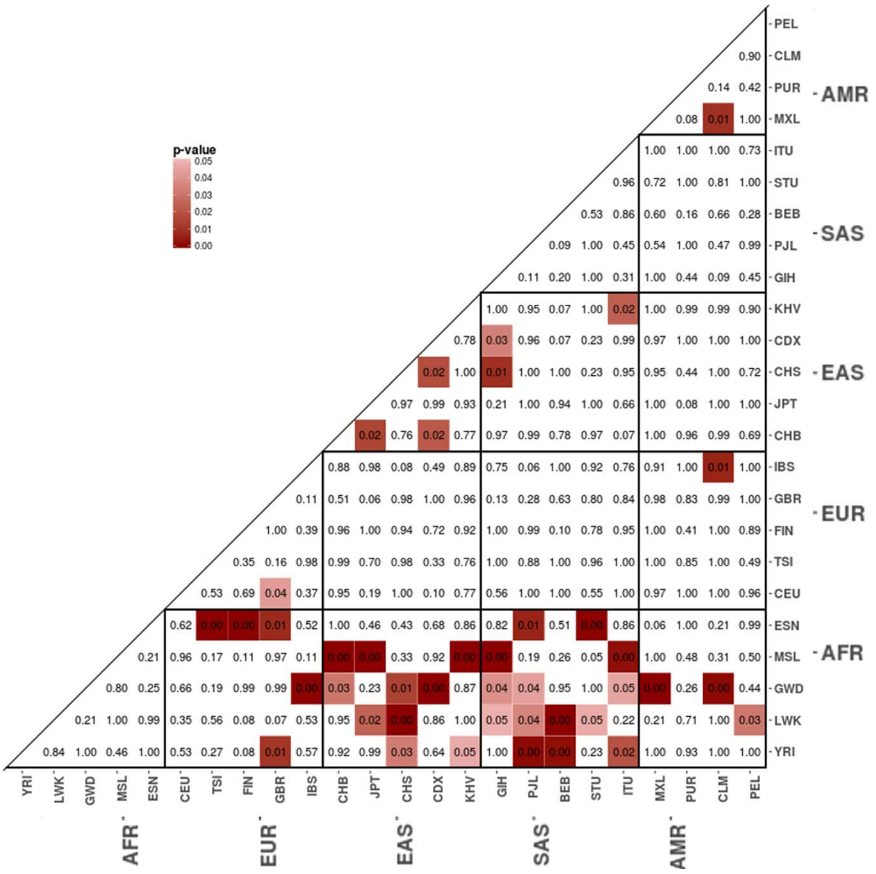

Figure S6. ZTGs present higher differentiation than randomly matched genes in African versus non-African population comparisons when comparing the proportion of highly differentiated SNPs. (a) For each comparison between geographical regions in the 1000GP dataset, p-values of the corresponding permutation test when comparing the proportion of highly differentiated SNPs of the whole set of 24 ZTGs with that of 10,000 resamplings of 24 genome-wide matched genes. (b) For each population comparison in the 1000GP dataset, p-values of the corresponding permutation test when comparing the proportion of highly differentiated SNPs of the whole set of 24 ZTGs with that of 10,000 resamplings of 24 genome-wide matched genes.

a

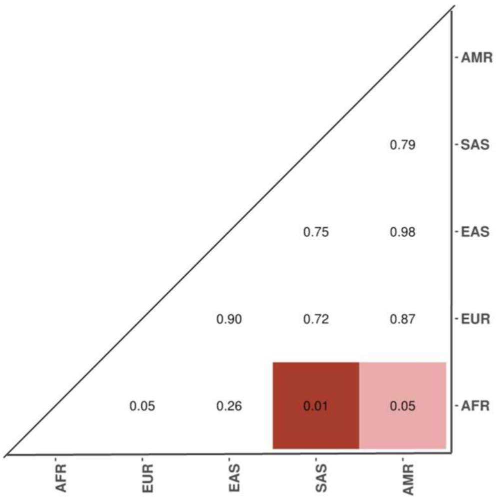
AMR
SAS
EAS
EUR
AFR
0.79
0.75 0.98
0.90 0.72 0.87
0.05 0.26 0.01 0.05
AFR
EUR
EAS
SAS
AMR

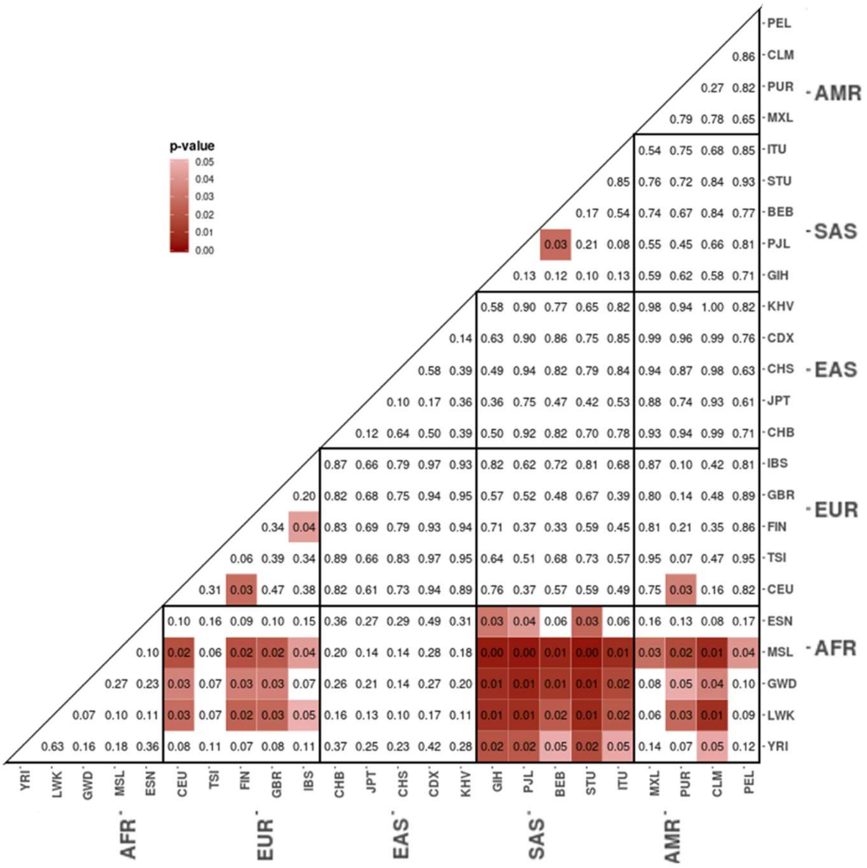
p-value
0.05
0.04
0.03
0.02
0.01
0.00
PEL
CLM
PUR
MXL
ITU
STU
BEB
PJL
GIH
KHV
CDX
CHS
JPT
CHB
IBS
GBR
FIN
TSI
CEU
ESN
MSL
GWD
LWK
YRI
AMR
SAS
EAS
EUR
AFR

b

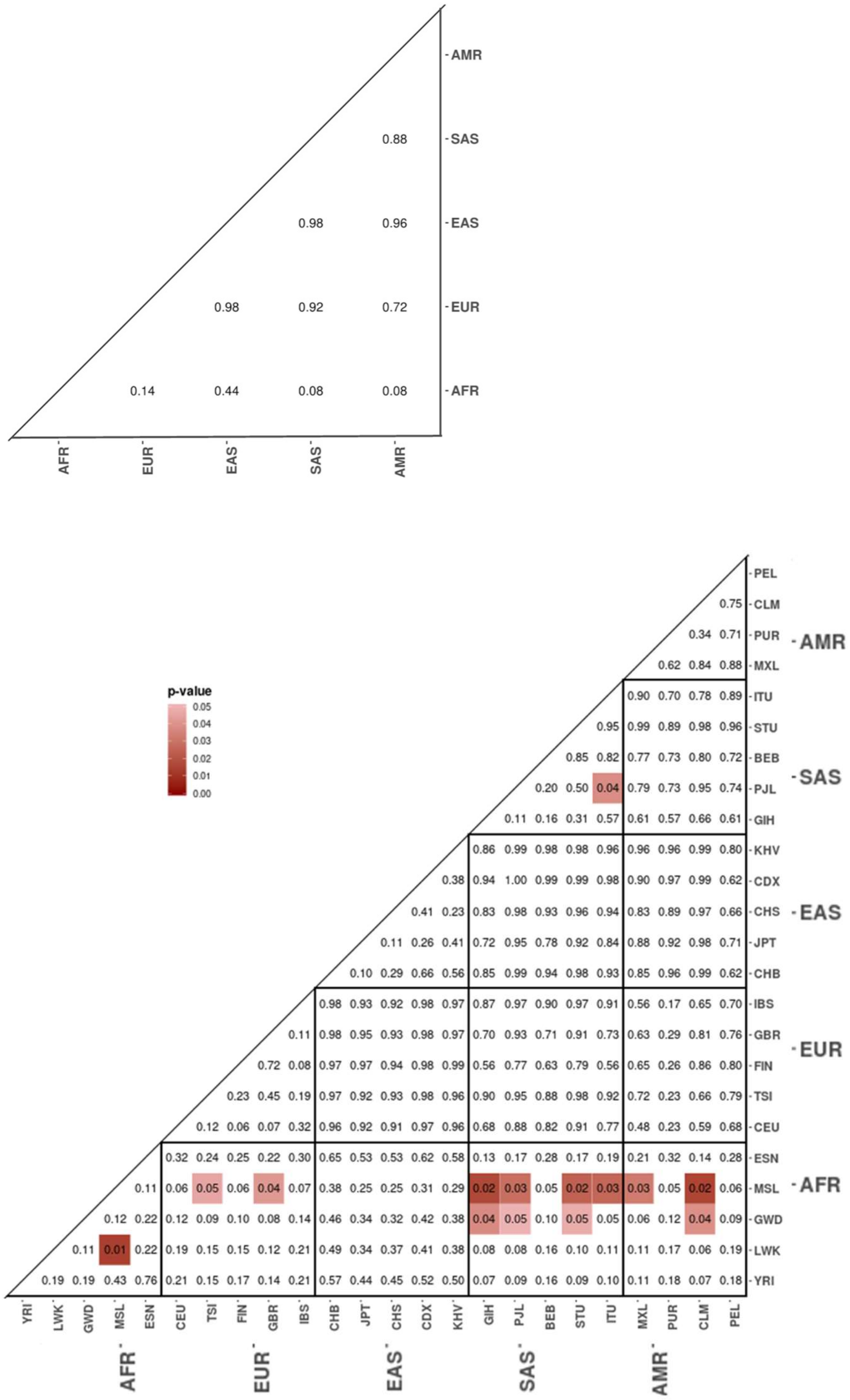
AMR
SAS 0.88
EAS 0.98 0.96
EUR 0.98 0.92 0.72
AFR 0.14 0.44 0.08 0.08
AFR EUR EAS SAS AMR
p-value
0.05
0.04
0.03
0.02
0.01
0.00
PEL
CLM 0.75
PUR 0.34 0.71
MXL 0.62 0.84 0.88
ITU 0.90 0.70 0.78 0.89
STU 0.95 0.99 0.89 0.98 0.96
BEB 0.85 0.82 0.77 0.73 0.80 0.72
PJL 0.20 0.50 0.04 0.79 0.73 0.95 0.74
GIH 0.11 0.16 0.31 0.57 0.61 0.57 0.66 0.61
KHV 0.86 0.99 0.98 0.98 0.96 0.96 0.96 0.99 0.80
CDX 0.38 0.94 1.00 0.99 0.99 0.98 0.90 0.97 0.99 0.62
CHS 0.41 0.23 0.83 0.98 0.93 0.96 0.94 0.83 0.89 0.97 0.66
JPT 0.11 0.26 0.41 0.72 0.95 0.78 0.92 0.84 0.88 0.92 0.98 0.71
CHB 0.10 0.29 0.66 0.56 0.85 0.99 0.94 0.98 0.93 0.85 0.96 0.99 0.62
IBS 0.98 0.93 0.92 0.98 0.97 0.87 0.97 0.90 0.97 0.91 0.56 0.17 0.65 0.70
GBR 0.11 0.98 0.95 0.93 0.98 0.97 0.70 0.93 0.71 0.91 0.73 0.63 0.29 0.81 0.76
FIN 0.72 0.08 0.97 0.97 0.94 0.98 0.99 0.56 0.77 0.63 0.79 0.56 0.65 0.26 0.86 0.80
TSI 0.23 0.45 0.19 0.97 0.92 0.93 0.98 0.96 0.90 0.95 0.88 0.98 0.92 0.72 0.23 0.66 0.79
CEU 0.12 0.06 0.07 0.32 0.96 0.92 0.91 0.97 0.96 0.68 0.88 0.82 0.91 0.77 0.48 0.23 0.59 0.68
ESN 0.32 0.24 0.25 0.22 0.30 0.65 0.53 0.53 0.62 0.58 0.13 0.17 0.28 0.17 0.19 0.21 0.32 0.14 0.28
MSL 0.11 0.06 0.05 0.06 0.04 0.07 0.38 0.25 0.25 0.31 0.29 0.02 0.03 0.05 0.02 0.03 0.03 0.05 0.02 0.06
GWD 0.12 0.22 0.12 0.09 0.10 0.08 0.14 0.46 0.34 0.32 0.42 0.38 0.04 0.05 0.10 0.05 0.05 0.06 0.12 0.04 0.09
LWK 0.11 0.01 0.22 0.19 0.15 0.15 0.12 0.21 0.49 0.34 0.37 0.41 0.38 0.08 0.08 0.16 0.10 0.11 0.11 0.17 0.06 0.19
YRI 0.19 0.19 0.43 0.76 0.21 0.15 0.17 0.14 0.21 0.57 0.44 0.45 0.52 0.50 0.07 0.09 0.16 0.09 0.10 0.11 0.18 0.07 0.18
YRI LWK GWD MSL ESN CEU TSI FIN GBR IBS CHB JPT CHS CDX KHV GIH PJL BEB STU ITU MXL PUR CLM PEL
AFR EUR EAS SAS AMR

c

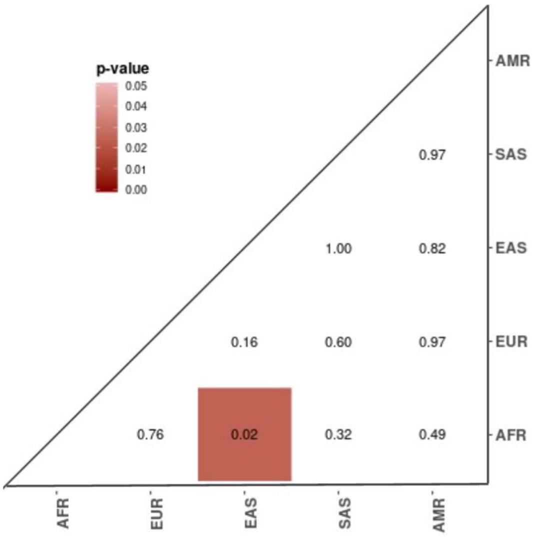

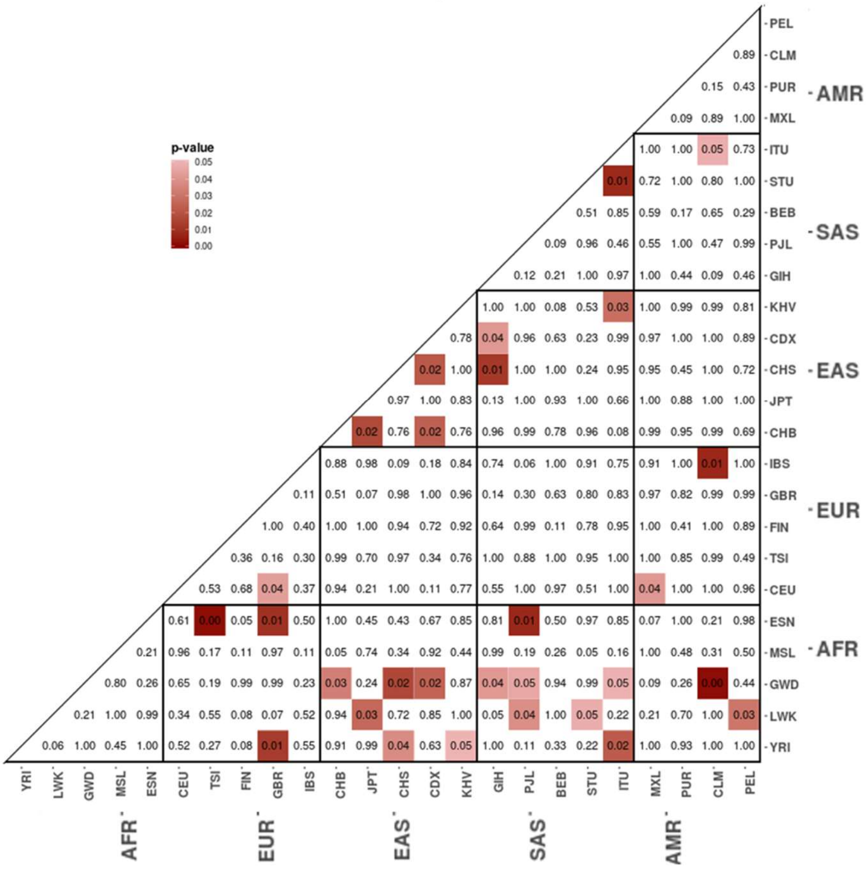

Figure S7. The unusual African versus non-African differentiation of the whole set of ZTGs mostly remains after subtracting the most highly differentiated ZTG (*SLC30A9*). For each F_{ST} population and geographical region comparison in the 1000GP dataset, p-values of the corresponding rank test when comparing the average F_{ST} value of the remaining set of 23 ZTGs with that of 10,000 resamplings of 23 genome-wide matched genes are shown. (a) Analysis when using the average of the Max F_{ST} per gene. (b) Analysis when using the average of the WA F_{ST} per gene. (c) For each population and geographical comparison in the 1000GP dataset, p-values of the corresponding permutation test when comparing the proportion of highly differentiated SNPs of the remaining set of 23 ZTGs with that of 10,000 resamplings of 23 genome-wide matched genes.

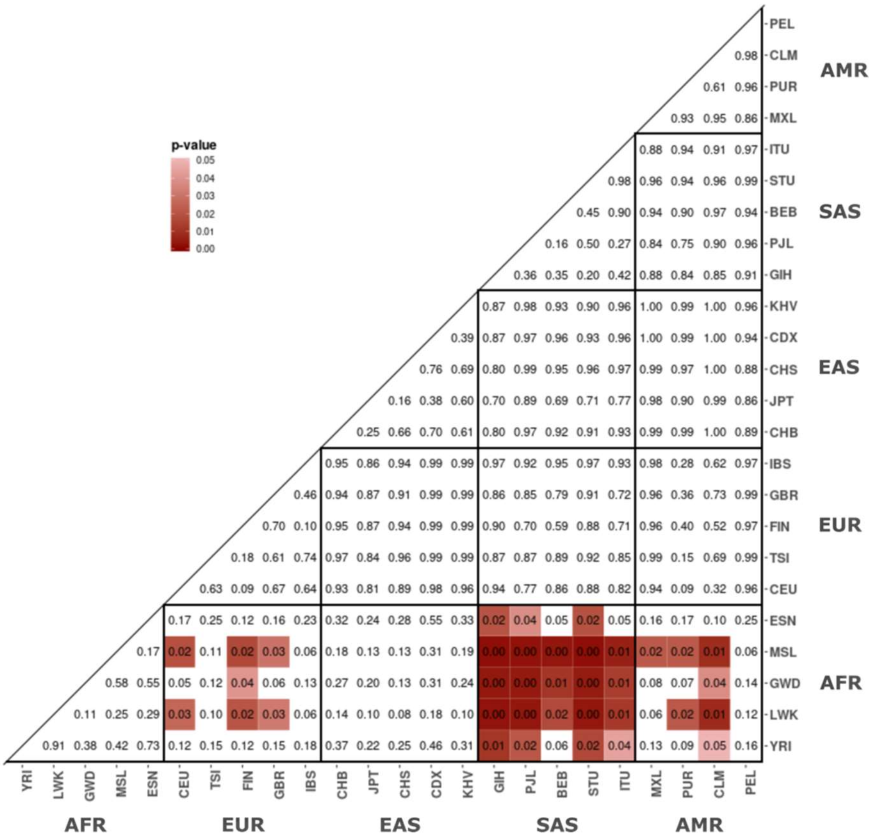

Figure S8. ZTGs present higher differentiation than randomly matched genes in African versus non-African population comparisons when aggregating the max F_{ST} scores per gene with the SUMSTAT statistic. For each F_{ST} population comparison in the 1000GP dataset, p-values of the corresponding rank test when comparing the sum of the Max F_{ST} value of the whole set of 24 ZTGs with that of 10,000 resamplings of 24 genome-wide matched genes with the SUMSTAT statistic while further controlling for SNP density.

Supplementary Note 1

Besides exploring for unusual patterns of variation on the whole set ZTGs, we investigated whether some individual ZTGs were recurrently identified as outliers across the F_{ST}, XP-EHH, iHS, and Tajima's D analyses when compared to reference genes matched for gene length, recombination, and gene content (Supplementary Tables S5-7). Per each population comparison, we considered as extremely differentiated ZTGs those that presented WA F_{ST} (or max-F_{ST}) values above the 99th percentile in the global distribution of WA F_{ST} (or max-F_{ST}) values obtained from the 5,146 reference genes for the same comparison. Similarly, to identify those individual ZTGs contributing more to the potential signals of selection detected for the whole set of 24 ZTGs, we ranked the mean gene value obtained for each statistic (iHS, XP-EHH, and Tajima's D) and population across the 5,146 genome-wide reference genes and identified as putative outliers those ZTGs within the top 1% percentile of the corresponding statistic distribution tail indicative of positive selection. Out of the 24 human ZTGs, 6 of them were found to display consistent patterns of variation indicative of strong positive selection across several populations in distinct geographical regions:

SLC30A9 is the gene that contributes most to the African versus non-African differentiation of ZTGs being detected as a consistent max-F_{ST} and WA F_{ST} top 1% outlier in the two datasets. It was also identified as a top 5% outlier for Avg XP-EHH in CHB (when using either YRI or CEU as reference population) as well as for max XP-EHH in CHB, GIH, and MXL (when using YRI as reference). Notably, all the population groups of the South Asian dataset and several populations in EUR, SAS, and EAS displayed *SLC30A9* as a top 5% outlier for Avg iHS. Moreover, it was also detected as a top 1% outlier for max-iHS in most South Asian groups. Finally, the Tajima's D values for *SLC30A9* in CHB and CDX were -2.54 and -2.46, which fall within the corresponding top 1% negative values of the empirical distribution obtained from genome-wide matched genes.

SLC39A5 was found as a consistent WA F_{ST} top 1% outlier in several African versus non-African pairwise comparisons of the 1000GP dataset, particularly when comparing SAS to AFR, as well as when comparing the population groups of the South Asian dataset to YRI. Moreover, the Tajima's D value of *SLC39A5* was not only detected as a top 1% outlier in the PJL, STU, and ITU populations but also as a top 5% outlier in several EUR, EAS, and AMR populations of the 1000GP dataset. Furthermore, it was also found within the top 5% outlier values for Avg XP-EHH in GIH and MXL when using YRI as reference.

SLC39A7 was detected as a consistent top 1% outlier mostly in Africa across several analyses of positive selection. In particular, the Tajima's D values obtained for *SLC39A7* in GWD, as well as

when analyzing all AFR populations together, fall within the corresponding top 1% negative values of the empirical distribution obtained from genome-wide reference genes. In addition, the LWK population also displayed an Avg iHS value for *SLC39A7* among the corresponding top 1% iHS scores. Moreover, when extending the empirical significance threshold to the top 5% values, *SLC39A7* appeared among the top signals of the Tajima's D test in all the remaining African populations of the 1000GP dataset (YRI, LWK, MSL, and ESN), as well as in the Avg iHS and Avg XP-EHH analyses of the YRI population (the latter when using either CEU, GIH or MXL as reference).

SLC39A8 was detected within the top 1% max XP-EHH values in CHB and GIH as well as within the top 5% max XP-EHH values of CEU and MXL when using YRI as reference. The signal in CHB was also found among the top 5% Avg XP-EHH values when using MXL, CEU, and GIH as reference populations. Moreover, *SLC39A8* was consistently detected among the top 5% max F_{ST} values when comparing the T-DR, T-TB, BEB, and STU populations of the South Asian dataset to the YRI.

SLC39A11 was identified as a consistent top 1% outlier for max F_{ST} mostly when comparing the South Asian populations of the 1000GP dataset to populations from both Europe and Africa. The same genetic differentiation pattern was observed in the South Asian dataset, where *SLC39A11* was detected within the top 5% max F_{ST} values in most population groups when compared to YRI. In addition, *SLC39A11* was detected within the top 1% iHS values in three South Asian populations of the 1000GP dataset (PJL, BEB and ITU) but also within the top 5% max iHS values in several populations from Africa, Europe, America, and South Asia. Furthermore, when analyzing the South Asian dataset, *SLC39A11* was found within the top 1% max XP-EHH values in all non-tribal South Asian groups, and within the top 5% max XP-EHH values in some tribal populations. In the 1000GP dataset, *SLC39A11* was consistently detected within the top 5% max XP-EHH values of the GIH population when using different populations as reference.

SLC39A4 was found within the top 1% max F_{ST} values in all African versus non-African population comparisons. This unusual pattern of differentiation is caused by the extreme allele frequencies differences observed in a non-synonymous substitution (rs1871534): the derived allele is found at high frequencies in Africa, whereas the ancestral one is almost fixed in most non-African populations. Although no other consistent signatures of strong positive selection are found accompanying this extremely differentiated non-synonymous SNP, *SLC39A4* was also observed within the top 1% Avg XP-EHH values in the GIH population of the 1000GP dataset (when using CEU as reference population) and within the top 5% Avg iHS values in most population groups of the South Asian dataset.
